# Author Correction: Injured epithelial cell states impact kidney allograft survival after T-cell-mediated rejection

**DOI:** 10.1038/s41467-026-73043-x

**Published:** 2026-05-11

**Authors:** Anna Maria Pfefferkorn, Lorenz Jahn, Patrick T. Gauthier, Vera Anna Kulow, Johannes Roeles, Niklas Müller-Bötticher, Louisa M. S. Gerhardt, Janna Leiz, Sadia Sarfraz, Izabela Plumbom, Robert Greite, Svjetlana Lovric, Jaba Gamrekelashvili, Florian Limbourg, Jessica Schmitz, Jan Hinrich Bräsen, Irina Scheffner, Igor M. Sauer, Felix Aigner, Janine Altmüller, Thomas Conrad, Wilfried Gwinner, Naveed Ishaque, Michael Fähling, Kai M. Schmidt-Ott, Philip F. Halloran, Muhammad Imtiaz Ashraf, Christian Hinze

**Affiliations:** 1 Department of Surgery, Experimental Surgery, Charité – Universitätsmedizin Berlin, corporate member of Freie Universität Berlin, Humboldt-Universität zu Berlin and Berlin Institute of Health, Berlin, Germany; 2 Department of Nephrology and Hypertension, Hannover Medical School, Hannover, Germany; 3 Alberta Transplant Applied Genomics Centre, Edmonton, AB Canada; 4 Institute of Translational Physiology, Charité – Universitätsmedizin Berlin, corporate member of Freie Universität Berlin and Humboldt-Universität zu Berlin, Berlin, Germany; 5 PRACTIS Clinician Scientist Program, Dean’s Office for Academic Career Development, Hannover Medical School, Hannover, Germany; 6 Berlin Institute of Health at Charité – Universitätsmedizin Berlin, Center of Digital Health, Berlin, Germany; 7 Freie Universität Berlin, Department of Mathematics and Computer Science, Berlin, Germany; 8 Department of Medicine V, University Medical Centre Mannheim, University of Heidelberg, Mannheim, Germany; 9 Genomics Technology Platform, Max Delbrück Center for Molecular Medicine, Berlin, Germany; 10 Core Unit Genomics, Berlin Institute of Health at Charité, Berlin, Germany; 11 Nephropathology Unit, Institute of Pathology, Hannover Medical School, Hannover, Germany; 12 Department of Surgery, Krankenhaus der Barmherzigen Brüder, Graz, Austria; 13 Department of Medicine, Division of Nephrology and Transplant Immunology, University of Alberta, Edmonton, AB Canada

**Keywords:** Kidney diseases, Nephrons

Correction to: *Nature Communications* 10.1038/s41467-026-68397-1, published online 28 January 2026

In the version of this article initially published, there were errors in the Fig. 8 color key, where the current labels (gray: “NR, Low,” black: “NR, high”; and light red: “TCMR, low,” red: “TCMR, high”) were originally interchanged. The figure is now amended in the HTML and PDF versions of the article.

