# Supplementary material for: Injured epithelial cell states impact kidney allograft survival after T-cell-mediated rejection
Source: Nat Commun. 2026 Jan 28;17:1060. doi: 10.1038/s41467-026-68397-1 (PMC12852170; doi:10.1038/s41467-026-68397-1)
Supplement: Supplementary file 1 — Supplemental Figures and Supplemental Table S1 [file 41467_2026_68397_MOESM1_ESM.pdf]

Supplemental material for

Injured epithelial cell states impact kidney allograft survival after T-cell-mediated rejection

Anna Maria Pfefferkorn[1,*], Lorenz Jahn[2,*], Patrick T. Gauthier[3,4,*], Vera Anna Kulow[5], Johannes Roeles[2,6], Niklas Müller-Bötticher[7,8], Louisa M. S. Gerhardt[9], Janna Leiz[2], Sadia Sarfraz[1], Izabela Plumbom[10,11], Robert Greite[2], Svjetlana Lovric[2], Jaba Gamrekelashvili[2], Florian Limbourg[2], Jessica Schmitz[12], Jan Hinrich Bräsen[12], Irina Scheffner[2], Igor M. Sauer[1], Felix Aigner[1,13], Janine Altmüller[10,11], Thomas Conrad[10,11], Wilfried Gwinner[2], Naveed Ishaque[7], Michael Fähling[5], Kai M. Schmidt-Ott[2], Philip F. Halloran[3,4,#], Muhammad Imtiaz Ashraf[1,#], Christian Hinze[2,#]

1 Department of Surgery, Experimental Surgery, Charité – Universitätsmedizin Berlin, corporate member of Freie Universität Berlin, Humboldt-Universität zu Berlin and Berlin Institute of Health, Berlin, Germany

2 Department of Nephrology and Hypertension, Hannover Medical School, Hannover, Germany

3 Alberta Transplant Applied Genomics Centre, Edmonton, Alberta, Canada

4 Department of Medicine, Division of Nephrology and Transplant Immunology, University of Alberta, Edmonton, Alberta, Canada.

5 Institute of Translational Physiology, Charité – Universitätsmedizin Berlin, corporate member of Freie Universität Berlin and Humboldt-Universität zu Berlin, Berlin, Germany

6 PRACTIS Clinician Scientist Program, Dean's Office for Academic Career Development, Hannover Medical School, Germany

7 Berlin Institute of Health at Charité – Universitätsmedizin Berlin, Center of Digital Health, Berlin, Germany

8 Freie Universität Berlin, Department of Mathematics and Computer Science, Berlin, Germany

9 Department of Medicine V, University Medical Centre Mannheim, University of Heidelberg, Mannheim, Germany

10 Genomics Technology Platform, Max Delbrück Center for Molecular Medicine, Berlin, Germany

11 Core Unit Genomics, Berlin Institute of Health at Charité, Berlin, Germany

12 Nephropathology Unit, Institute of Pathology, Hannover Medical School, Hannover, Germany

13 Department of Surgery, Krankenhaus der Barmherzigen Brüder, Graz, Austria

* These authors contributed equally

# These authors jointly supervised this work

Corresponding authors:

Prof. Dr. Philip F. Halloran, Email: phallora@ualberta.ca

Dr. Muhammad Imtiaz Ashraf, Email: muhammad-imtiaz.ashraf@charite.de

Prof. Dr. Christian Hinze, Email: hinze.christian@mh-hannover.de

A

							Banff scoring		
**Group**	**Tx combination**	**Mouse IDs**	**Sample name in manuscript (if applicable)**	**Used for clinical anaylses (creatinine etc.)**	**snRNA-seq**	**ST**	**i**	**t**	**v**
SYN		M1	C57BL/6 to C57BL/6 1	x	x	x	0	0	0
SYN	C57BL/6 - C57BL/6	M2		x			0	0	0
SYN		M3		x			0	0	0
SYN		M1	BALB/c to BALB/c 1	x	x	x	0	0	0
SYN	Balb/c - Balb/c	M2	BALB/c to BALB/c 2	x	x		0	0	0
SYN		M3		x			0	0	0
ALLO		M1	BALB/c to C57BL/6 1	x	x	x	2	1	1
ALLO	Balb/c - C57BL/6	M2		x			2	2	1
ALLO		M3	BALB/c to C57BL/6 3	x	x	x	3	1	1
ALLO		M1	C57BL/6 to BALB/c 1	x	x	x	3	1	1
ALLO	C57BL/6 - Balb/c	M2	C57BL/6 to BALB/c 2	x	x	x	3	3	1
ALLO		M3	C57BL/6 to BALB/c 3	x	x	x	3	2	1
ALLO		M4		x			3	2	1

B

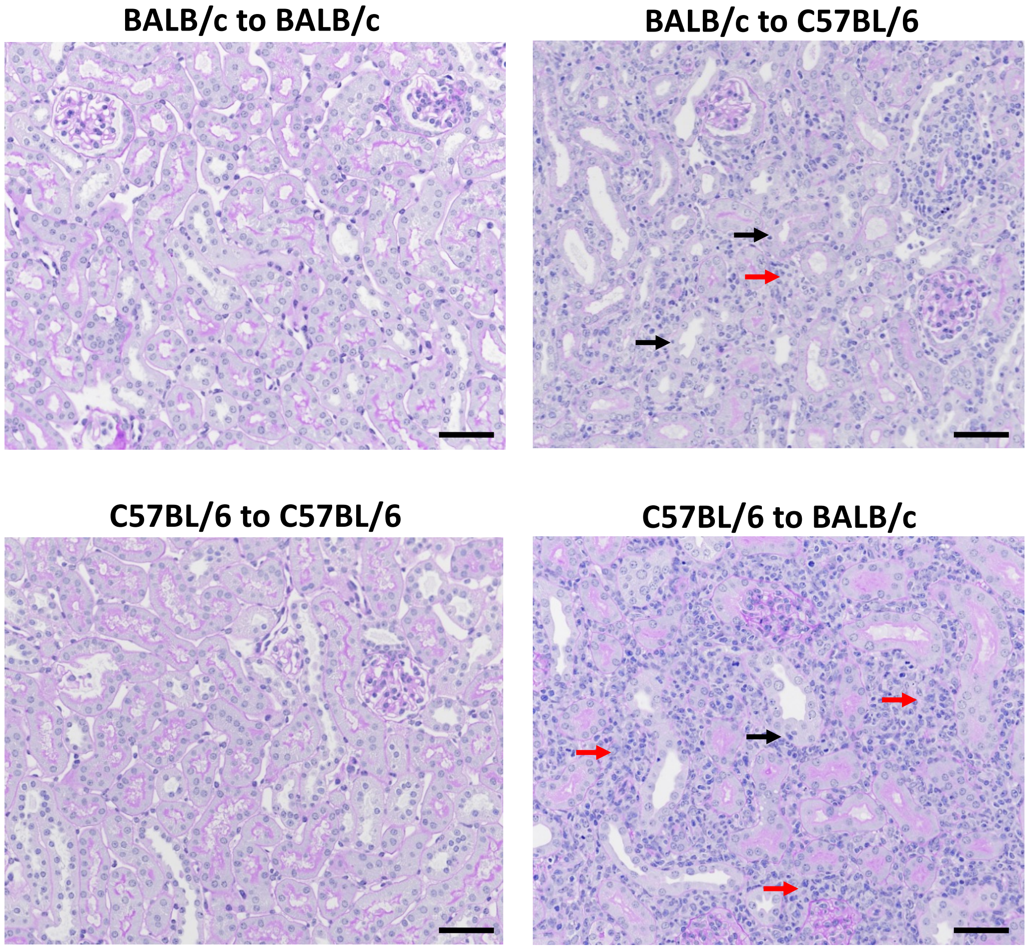

Supplemental Figure S1: Sample information and histology of mouse kidneys from syngeneic and allogeneic groups. A. *Sample information for mouse kidney samples.* ***B.*** *Representative periodic acid-Schiff (PAS) stained images of the BALB/c and C57BL/6 kidney*

isografts and the kidney allografts BALB/c to C57BL/6, C57BL/6 to BALB/c at post-operative day 7, showing interstitial inflammation (red arrows) and tubulitis (black arrows). Note that part of the C57BL/6 to BALB/c allograft is depicted in Fig. 1 in the main body. Scale bar: 50 μm.

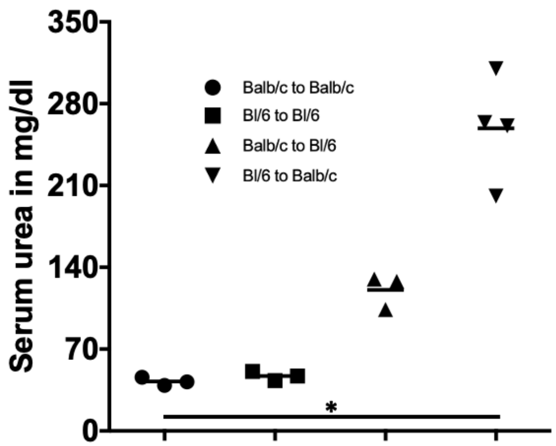

Supplemental Figure S2: Serum urea measurements from syngeneic and allogeneic mice. *Data were analyzed using the Kruskal–Wallis test followed by Dunn's multiple comparisons test (n = 3). Adjusted P-value: *<0.05. Source data are provided as a Source Data file.*

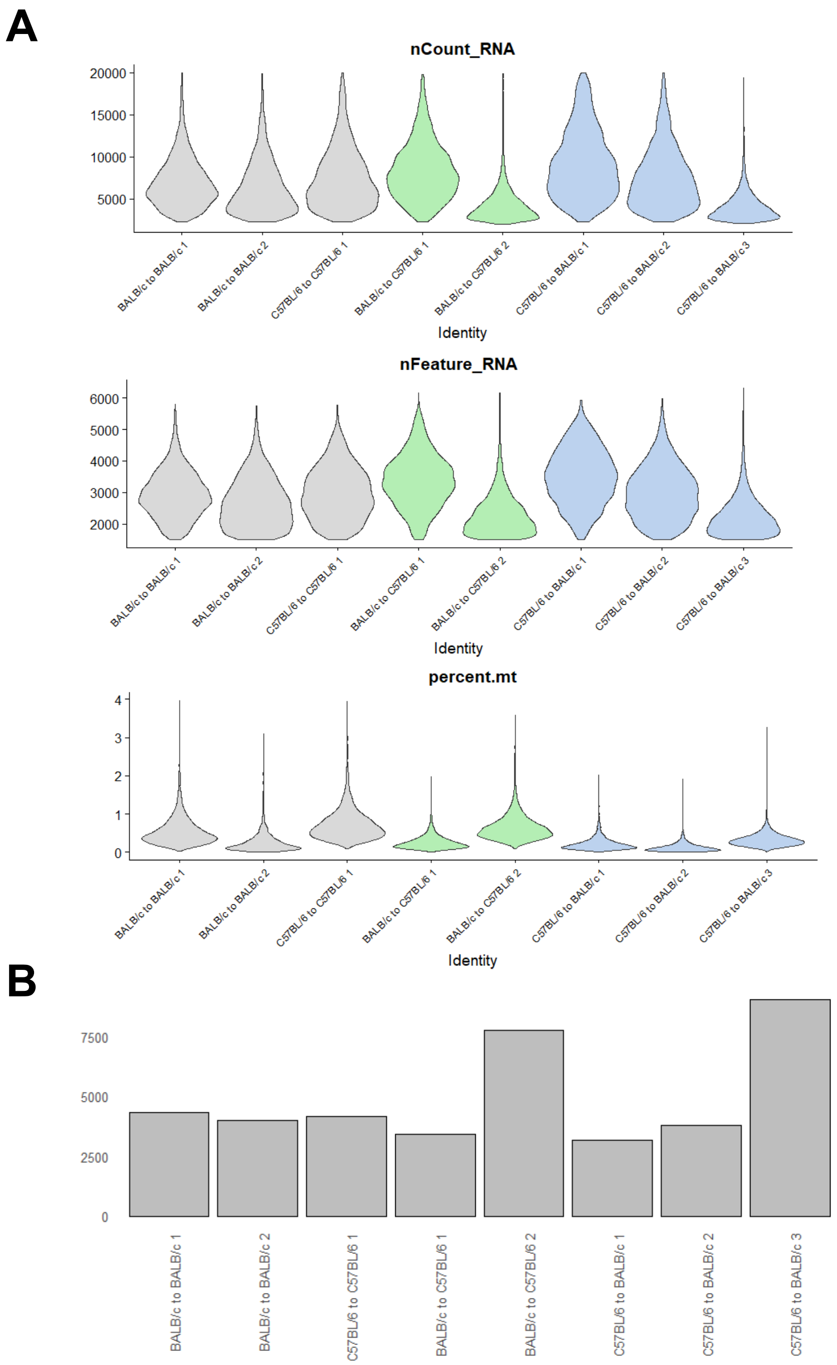

Supplemental Figure S3: Quality metrics for mouse snRNA-seq data. A. *Number of detected genes (nFeature_RNA), transcripts (nCount_RNA) and percentage of mitochondrial reads (percent.mt) per sample.* ***B.*** *Number of detected cells per sample. Source data are provided as a Source Data file.*

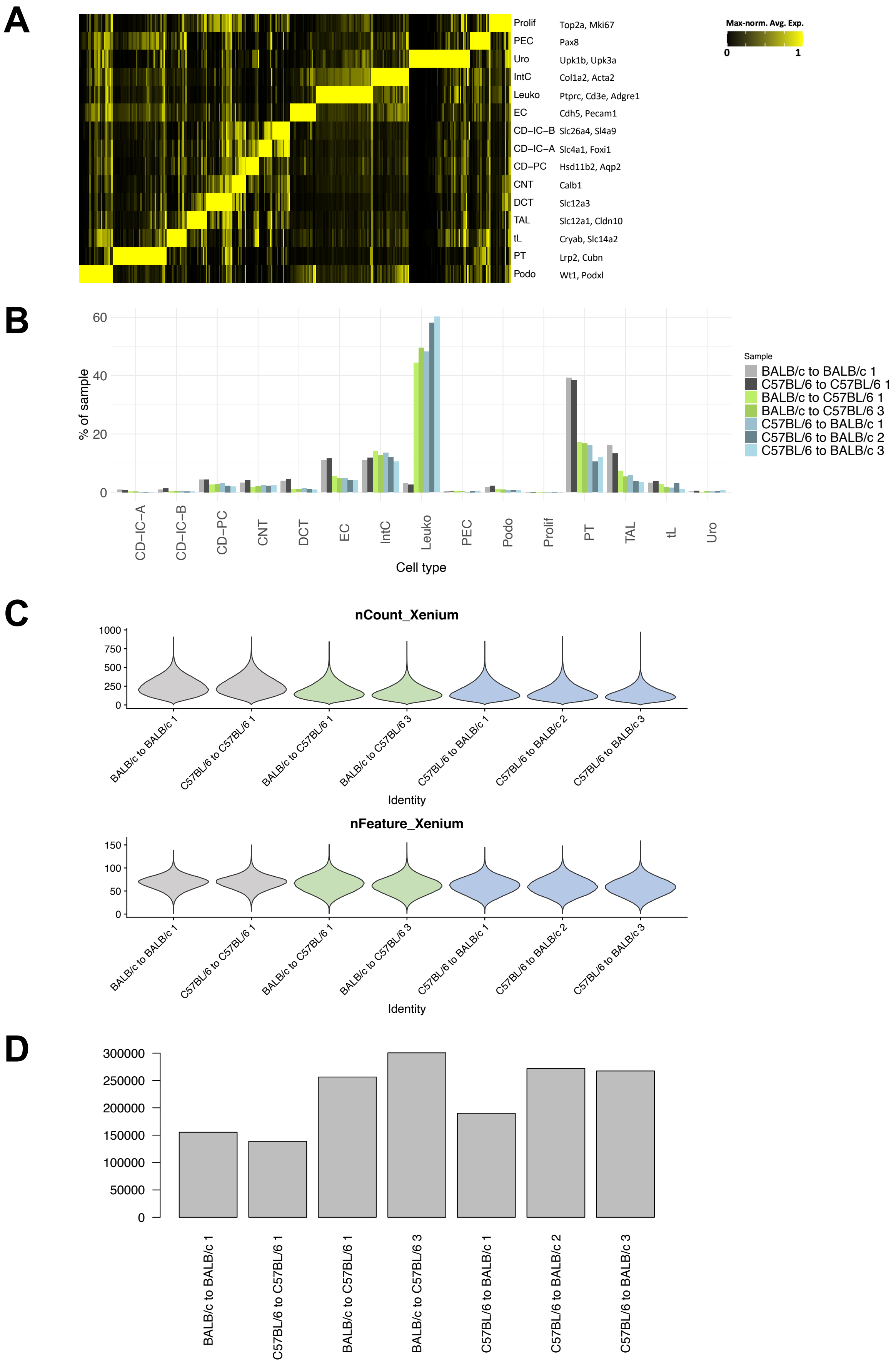
A
Prolif
PEC
Uro
IntC
Leuko
EC
CD−IC−B
CD−IC−A
CD−PC
CNT
DCT
TAL
tL
PT
Podo
Top2a, Mki67
Pax8
Upk1b, Upk3a
Col1a2, Acta2
Ptprc, Cd3e, Adgre1
Cdh5, Pecam1
Slc26a4, Sl4a9
Slc4a1, Foxi1
Hsd11b2, Aqp2
Calb1
Slc12a3
Slc12a1, Cldn10
Cryab, Slc14a2
Lrp2, Cubn
Wt1, Podxl
Max-norm. Avg. Exp.
0
1
B
% of sample
60
40
20
0
CD−IC−A
CD−IC−B
CD−PC
CNT
DCT
EC
IntC
Leuko
PEC
Podo
Prolif
PT
TAL
tL
Uro
Cell type
Sample
BALB/c to BALB/c 1
C57BL/6 to C57BL/6 1
BALB/c to C57BL/6 1
BALB/c to C57BL/6 3
C57BL/6 to BALB/c 1
C57BL/6 to BALB/c 2
C57BL/6 to BALB/c 3
C
nCount_Xenium
1000
750
500
250
0
BALB/c to BALB/c 1
C57BL/6 to C57BL/6 1
BALB/c to C57BL/6 1
BALB/c to C57BL/6 3
C57BL/6 to BALB/c 1
C57BL/6 to BALB/c 2
C57BL/6 to BALB/c 3
Identity
nFeature_Xenium
150
100
50
0
BALB/c to BALB/c 1
C57BL/6 to C57BL/6 1
BALB/c to C57BL/6 1
BALB/c to C57BL/6 3
C57BL/6 to BALB/c 1
C57BL/6 to BALB/c 2
C57BL/6 to BALB/c 3
Identity
D
300000
250000
200000
150000
100000
50000
0
BALB/c to BALB/c 1
C57BL/6 to C57BL/6 1
BALB/c to C57BL/6 1
BALB/c to C57BL/6 3
C57BL/6 to BALB/c 1
C57BL/6 to BALB/c 2
C57BL/6 to BALB/c 3

Supplemental Figure S4: Quality metrics and marker gene analysis for mouse ST data. A. *Broad cell type assignment was performed as detailed in the methods. The heatmap shows marker gene expression, with values normalized to the maximum average expression per gene. Conserved canonical cell type markers shown in the heatmap are displayed to the right of the cell type label.* ***B.*** *Relative abundances of major cell types in ST data per sample.* ***C.*** *Number of detected genes (nFeature_Xenium) and transcripts (nCount_Xenium) per sample.* ***D.*** *Number of detected and segmented cells per sample. Source data are provided as a Source Data file.*

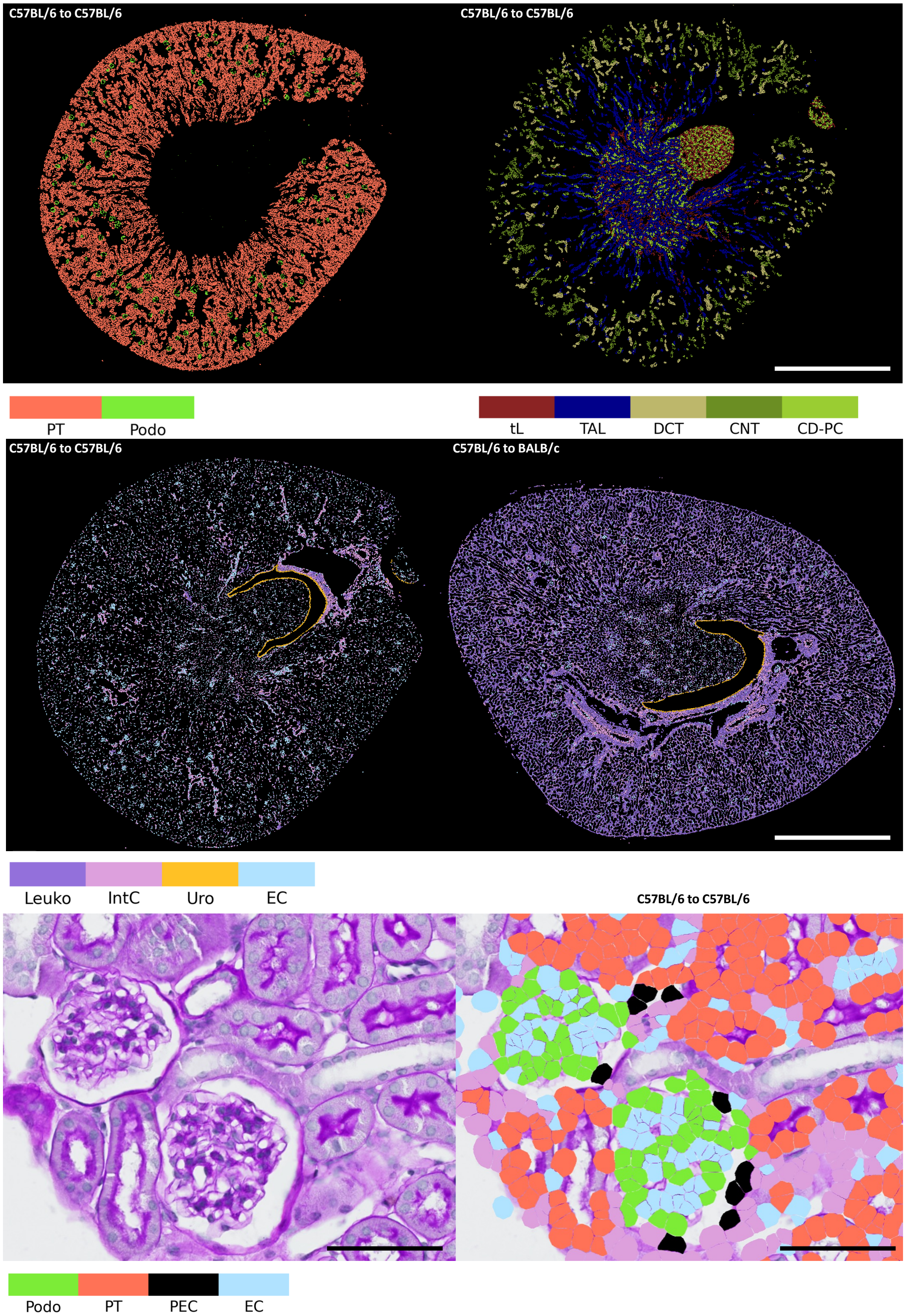

Supplemental Figure S5: Spatial distribution of major cell types. *Plots show, as indicated below the respective image, the distribution of major cell types within the kidney as determined*

from cell type label transfer from snRNA-seq data. Note the massive infiltration of the rejecting kidney with leukocytes (middle right panel). Modality of transplantation is shown in the upper left corner of the overview images. Scale bar overview: 1000 µm, scale bar lower images: 50 µm. All experiments repeated for all spatial transcriptomic samples with similar result.

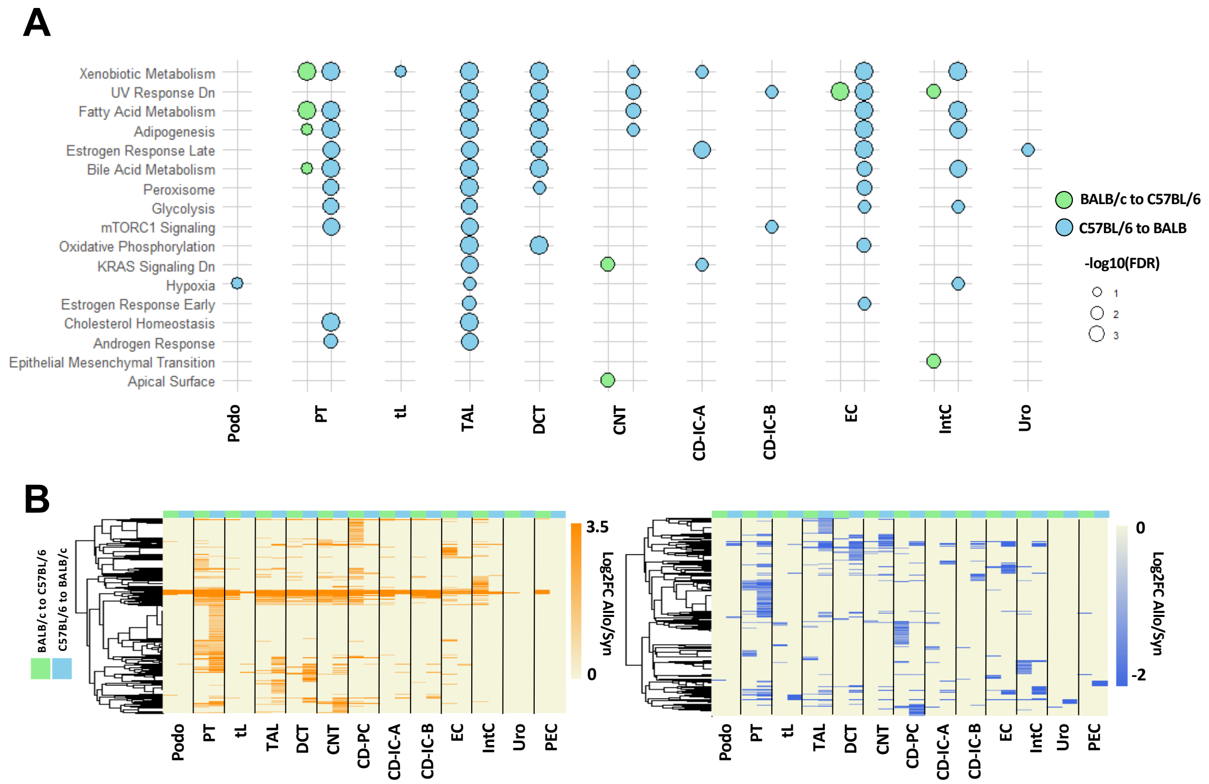

Supplemental Figure S6: Pathway enrichment analysis of differentially regulated genes. A. *Dot plot of pathway enrichment analysis of differentially downregulated genes for each major cell type and transplant group, e.g. BALB/c to C57BL/6 (green) and C57BL/6 to BALB/c (blue). Shown are all significant pathways (FDR < 0.05) from gene set enrichment analysis using hallmark gene sets.* ***B.*** *Heatmaps displaying differentially upregulated (left) and downregulated (right) genes by cell type for both groups. Heatmaps show log2 fold changes of allogeneic kidneys compared to their respective syngeneic controls. Source data are provided as a Source Data file.*

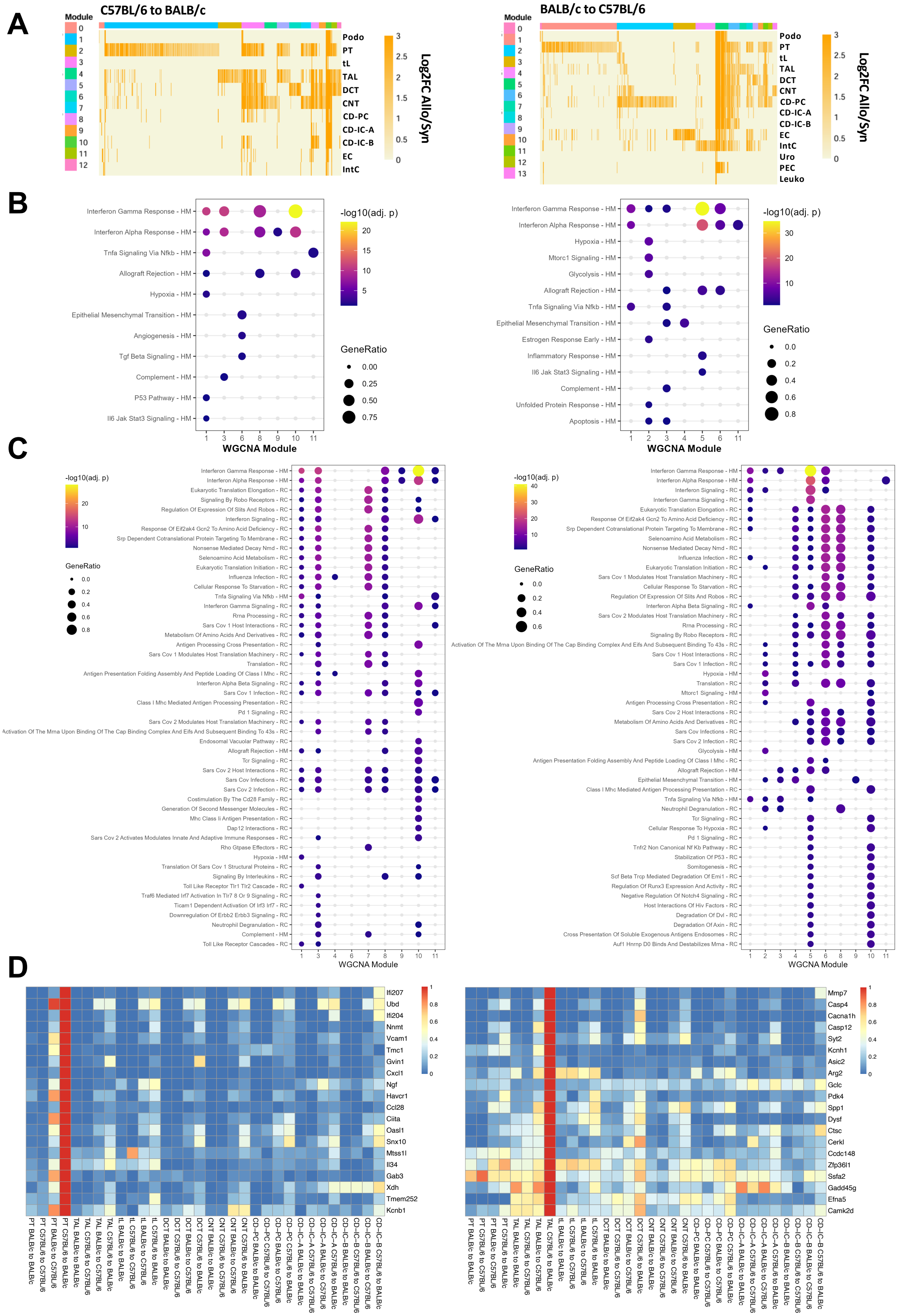

A
C57BL/6 to BALB/c
Module
0
1
2
3
4
5
6
7
8
9
10
11
12
Podo
PT
tL
TAL
DCT
CNT
CD-PC
CD-IC-A
CD-IC-B
EC
IntC
3
2.5
2
1.5
1
0.5
0
Log2FC Allo/Syn
BALB/c to C57BL/6
Module
0
1
2
3
4
5
6
7
8
9
10
11
12
13
Podo
PT
tL
TAL
DCT
CNT
CD-PC
CD-IC-A
CD-IC-B
EC
IntC
Uro
PEC
Leuko
Log2FC Allo/Syn
B
Interferon Gamma Response - HM
Interferon Alpha Response - HM
Tnfa Signaling Via Nfkb - HM
Allograft Rejection - HM
Hypoxia - HM
Epithelial Mesenchymal Transition - HM
Angiogenesis - HM
Tgf Beta Signaling - HM
Complement - HM
P53 Pathway - HM
Il6 Jak Stat3 Signaling - HM
1 3 6 8 9 10 11
-log10(adj. p)
20
15
10
5
GeneRatio
0.00
0.25
0.50
0.75
Interferon Gamma Response - HM
Interferon Alpha Response - HM
Hypoxia - HM
Mtorc1 Signaling - HM
Glycolysis - HM
Allograft Rejection - HM
Tnfa Signaling Via Nfkb - HM
Epithelial Mesenchymal Transition - HM
Estrogen Response Early - HM
Inflammatory Response - HM
Il6 Jak Stat3 Signaling - HM
Complement - HM
Unfolded Protein Response - HM
Apoptosis - HM
1 2 3 4 5 6 11
-log10(adj. p)
30
20
10
GeneRatio
0.0
0.2
0.4
0.6
0.8
WGCNA Module
C
-log10(adj. p)
20
10
GeneRatio
0.0
0.2
0.4
0.6
0.8
Interferon Gamma Response - HM
Interferon Alpha Response - HM
Eukaryotic Translation Elongation - RC
Signaling By Robo Receptors - RC
Regulation Of Expression Of Slits And Robos - RC
Interferon Signaling - RC
Response Of Eif2ak4 Gcn2 To Amino Acid Deficiency - RC
Srp Dependent Cotranslational Protein Targeting To Membrane - RC
Nonsense Mediated Decay Nmd - RC
Selenoamino Acid Metabolism - RC
Eukaryotic Translation Initiation - RC
Influenza Infection - RC
Cellular Response To Starvation - RC
Tnfa Signaling Via Nfkb - HM
Interferon Gamma Signaling - RC
Rrna Processing - RC
Sars Cov 1 Host Interactions - RC
Metabolism Of Amino Acids And Derivatives - RC
Antigen Processing Cross Presentation - RC
Sars Cov 1 Modulates Host Translation Machinery - RC
Translation - RC
Antigen Presentation Folding Assembly And Peptide Loading Of Class I Mhc - RC
Interferon Alpha Beta Signaling - RC
Sars Cov 1 Infection - RC
Class I Mhc Mediated Antigen Processing Presentation - RC
Pd 1 Signaling - RC
Sars Cov 2 Modulates Host Translation Machinery - RC
Activation Of The Mrna Upon Binding Of The Cap Binding Complex And Eifs And Subsequent Binding To 43s - RC
Endosomal Vacuolar Pathway - RC
Allograft Rejection - HM
Tcr Signaling - RC
Sars Cov 2 Host Interactions - RC
Sars Cov Infections - RC
Sars Cov 2 Infection - RC
Costimulation By The Cd28 Family - RC
Generation Of Second Messenger Molecules - RC
Mhc Class Ii Antigen Presentation - RC
Dap12 Interactions - RC
Sars Cov 2 Activates Modulates Innate And Adaptive Immune Responses - RC
Rho Gtpase Effectors - RC
Hypoxia - HM
Translation Of Sars Cov 1 Structural Proteins - RC
Signaling By Interleukins - RC
Toll Like Receptor Tlr1 Tlr2 Cascade - RC
Traf6 Mediated Irf7 Activation In Tlr7 8 Or 9 Signaling - RC
Ticam1 Dependent Activation Of Irf3 Irf7 - RC
Downregulation Of Erbb2 Erbb3 Signaling - RC
Neutrophil Degranulation - RC
Complement - HM
Toll Like Receptor Cascades - RC
1 3 4 6 7 8 9 10 11
WGCNA Module
-log10(adj. p)
40
30
20
10
GeneRatio
0.0
0.2
0.4
0.6
Interferon Gamma Response - HM
Interferon Alpha Response - HM
Interferon Signaling - RC
Interferon Gamma Signaling - RC
Eukaryotic Translation Elongation - RC
Response Of Eif2ak4 Gcn2 To Amino Acid Deficiency - RC
Srp Dependent Cotranslational Protein Targeting To Membrane - RC
Selenoamino Acid Metabolism - RC
Nonsense Mediated Decay Nmd - RC
Influenza Infection - RC
Eukaryotic Translation Initiation - RC
Sars Cov 1 Modulates Host Translation Machinery - RC
Cellular Response To Starvation - RC
Regulation Of Expression Of Slits And Robos - RC
Interferon Alpha Beta Signaling - RC
Sars Cov 2 Modulates Host Translation Machinery - RC
Rrna Processing - RC
Signaling By Robo Receptors - RC
Activation Of The Mrna Upon Binding Of The Cap Binding Complex And Eifs And Subsequent Binding To 43s - RC
Sars Cov 1 Host Interactions - RC
Sars Cov 1 Infection - RC
Hypoxia - HM
Translation - RC
Mtorc1 Signaling - HM
Antigen Processing Cross Presentation - RC
Sars Cov 2 Host Interactions - RC
Metabolism Of Amino Acids And Derivatives - RC
Sars Cov Infections - RC
Sars Cov 2 Infection - RC
Glycolysis - HM
Antigen Presentation Folding Assembly And Peptide Loading Of Class I Mhc - RC
Allograft Rejection - HM
Epithelial Mesenchymal Transition - HM
Class I Mhc Mediated Antigen Processing Presentation - RC
Tnfa Signaling Via Nfkb - HM
Neutrophil Degranulation - RC
Tcr Signaling - RC
Cellular Response To Hypoxia - RC
Pd 1 Signaling - RC
Tnfr2 Non Canonical Nf Kb Pathway - RC
Stabilization Of P53 - RC
Somitogenesis - RC
Scf Beta Trcp Mediated Degradation Of Emi1 - RC
Regulation Of Runx3 Expression And Activity - RC
Negative Regulation Of Notch4 Signaling - RC
Host Interactions Of Hiv Factors - RC
Degradation Of Dvl - RC
Degradation Of Axin - RC
Cross Presentation Of Soluble Exogenous Antigens Endosomes - RC
Auf1 Hnrnp D0 Binds And Destabilizes Mrna - RC
1 2 3 4 5 6 8 9 10 11
WGCNA Module
D
Ifi207
Ubd
Ifi204
Nnmt
Vcam1
Tmc1
Gvin1
Cxcl1
Ngf
Havcr1
Ccl28
Ciita
Oasl1
Snx10
Mtss1l
Il34
Gab3
Xdh
Tmem252
Kcnb1
1
0.8
0.6
0.4
0.2
0
PT BALB/c to BALB/c
PT C57BL/6 to C57BL/6
PT BALB/c to C57BL/6
PT C57BL/6 to BALB/c
TAL BALB/c to BALB/c
TAL C57BL/6 to C57BL/6
TAL BALB/c to C57BL/6
TAL C57BL/6 to BALB/c
tL BALB/c to BALB/c
tL C57BL/6 to C57BL/6
tL BALB/c to C57BL/6
tL C57BL/6 to BALB/c
DCT BALB/c to BALB/c
DCT C57BL/6 to C57BL/6
DCT BALB/c to C57BL/6
DCT C57BL/6 to BALB/c
CNT BALB/c to BALB/c
CNT C57BL/6 to C57BL/6
CNT BALB/c to C57BL/6
CNT C57BL/6 to BALB/c
CD−PC BALB/c to BALB/c
CD−PC C57BL/6 to C57BL/6
CD−PC BALB/c to C57BL/6
CD−PC C57BL/6 to BALB/c
CD−IC−A BALB/c to BALB/c
CD−IC−A C57BL/6 to C57BL/6
CD−IC−A BALB/c to C57BL/6
CD−IC−A C57BL/6 to BALB/c
CD−IC−B BALB/c to BALB/c
CD−IC−B C57BL/6 to C57BL/6
CD−IC−B BALB/c to C57BL/6
CD−IC−B C57BL/6 to BALB/c
Mmp7
Casp4
Cacna1h
Casp12
Syt2
Kcnh1
Asic2
Arg2
Gclc
Pdk4
Spp1
Dysf
Ctsc
Cerkl
Ccdc148
Zfp36l1
Ssfa2
Gadd45g
Efna5
Camk2d

Supplemental Figure S7: Analysis of cell type-specific modules and top differentially upregulated genes. A. *Heatmaps showing the distribution of differentially expressed genes (DEGs with same parameters as in Figure 2) across kidney cell types. Gene co-expression modules were defined using Weighted Gene Correlation Network Analysis (WGCNA) and are displayed with log2 fold-change (allogeneic samples versus respective syngeneic samples) from the DEG analysis.* ***B.*** *Hallmark pathway enrichment ($q < 0.05$) per WGCNA module. Dot color indicates -log10(adjusted p-value) and dot size reflects the gene ratio.* ***C.*** *Hallmark and Reactome pathway enrichment ($q < 0.1$) per WGCNA module, displayed as in B. to include more pathways.* ***D.*** *Heatmaps of top differentially upregulated genes in PT and TAL C57BL/6 to BALB/c versus C57BL/6 to C57BL/6. Source data are provided as a Source Data file.*

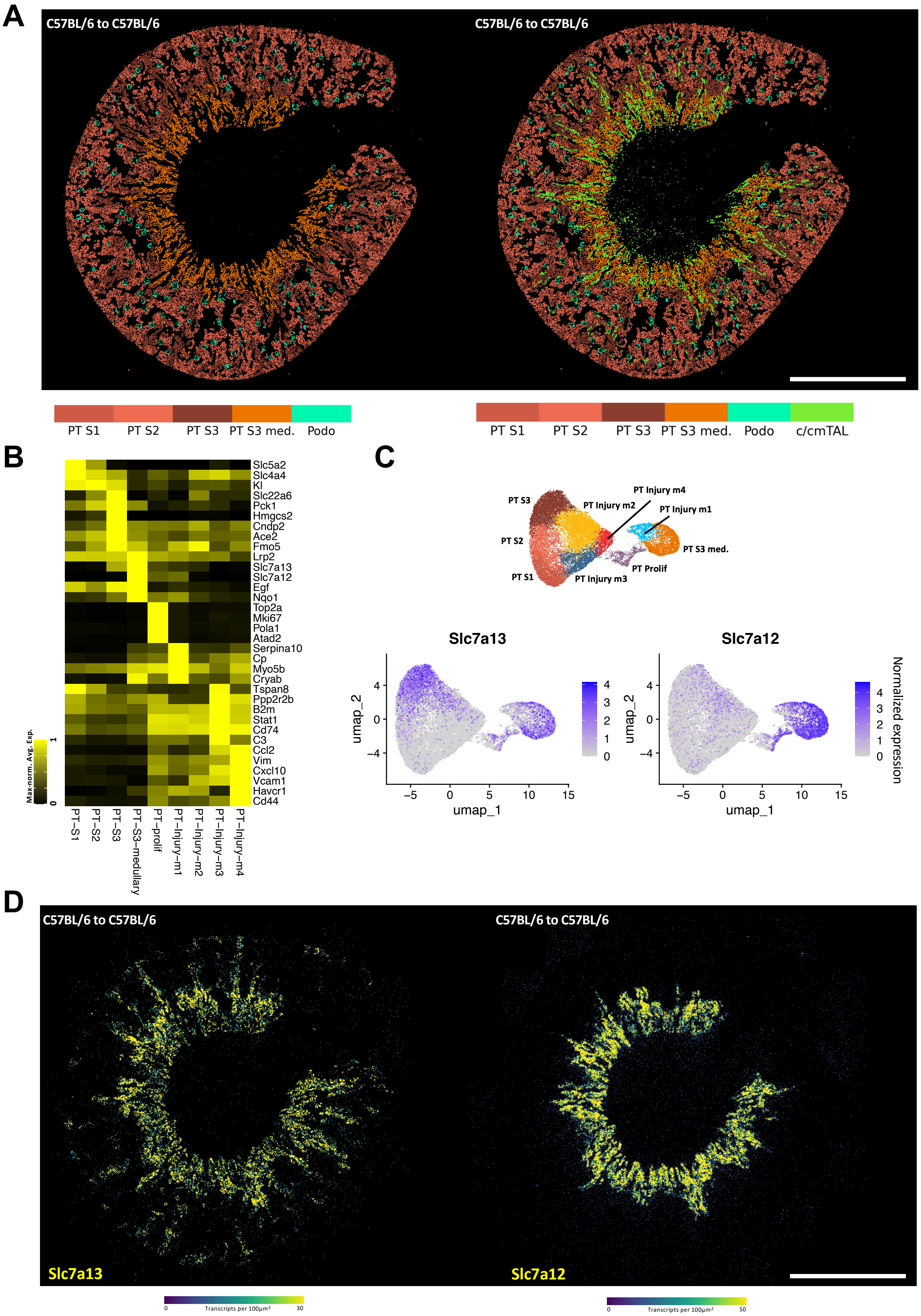
A
C57BL/6 to C57BL/6
C57BL/6 to C57BL/6
PT S1
PT S2
PT S3
PT S3 med.
Podo
PT S1
PT S2
PT S3
PT S3 med.
Podo
c/cmTAL
B
Slc5a2
Slc4a4
Kl
Slc22a6
Pck1
Hmgcs2
Cndp2
Ace2
Fmo5
Lrp2
Slc7a13
Slc7a12
Egf
Nqo1
Top2a
Mki67
Pola1
Atad2
Serpina10
Cp
Myo5b
Cryab
Tspan8
Ppp2r2b
B2m
Stat1
Cd74
C3
Ccl2
Vim
Cxcl10
Vcam1
Havcr1
Cd44
Max-norm. Avg. Exp.
1
0
PT−S1
PT−S2
PT−S3
PT−S3−medullary
PT−prolif
PT−Injury−m1
PT−Injury−m2
PT−Injury−m3
PT−Injury−m4
C
PT Injury m4
PT S3
PT Injury m2
PT Injury m1
PT S2
PT S3 med.
PT Prolif
PT S1
PT Injury m3
Slc7a13
Slc7a12
umap_2
umap_1
Normalized expression
D
C57BL/6 to C57BL/6
C57BL/6 to C57BL/6
Slc7a13
Slc7a12
Transcripts per 100µm²
Transcripts per 100µm²

Supplemental Figure S8: Spatial distribution of healthy PT segments from snRNA-seq label transfer. A. *Distribution of PT subtypes obtained by SingleR-based label transfer from snRNA-seq data. Podocytes are included to facilitate identification of the kidney cortex. While cells from the S3 medullary cluster localize strictly to the medulla, cells from the S3 cluster extend into the medullary rays reaching into the cortex. This visual impression is further emphasized by highlighting cortical and corticomedullary TAL segments in the right panel.* ***B.*** *Expression of snRNA-seq-derived PT marker genes in spatial transcriptomics data (see Fig. 3 for expression in snRNA-seq data).* ***C.*** *Feature plots of the S3 and S3 medullary marker gene Slc7a13 and the S3 medullary marker gene Slc7a12 in PT cells from snRNA-seq data.* ***D.*** *Transcript density plots for Slc7a12 and Slc7a13. Spatial distribution of S3 and S3 medullary cells corresponds closely to the expression domains of Slc7a13 and Slc7a12 in ST data as expected from label transfer and snRNA-seq data (compare to A.). Transcript densities are represented according to the color scale. Scale bar: 1000 µm. Source data are provided as a Source Data file. All experiments repeated for all spatial transcriptomic samples with similar result.*

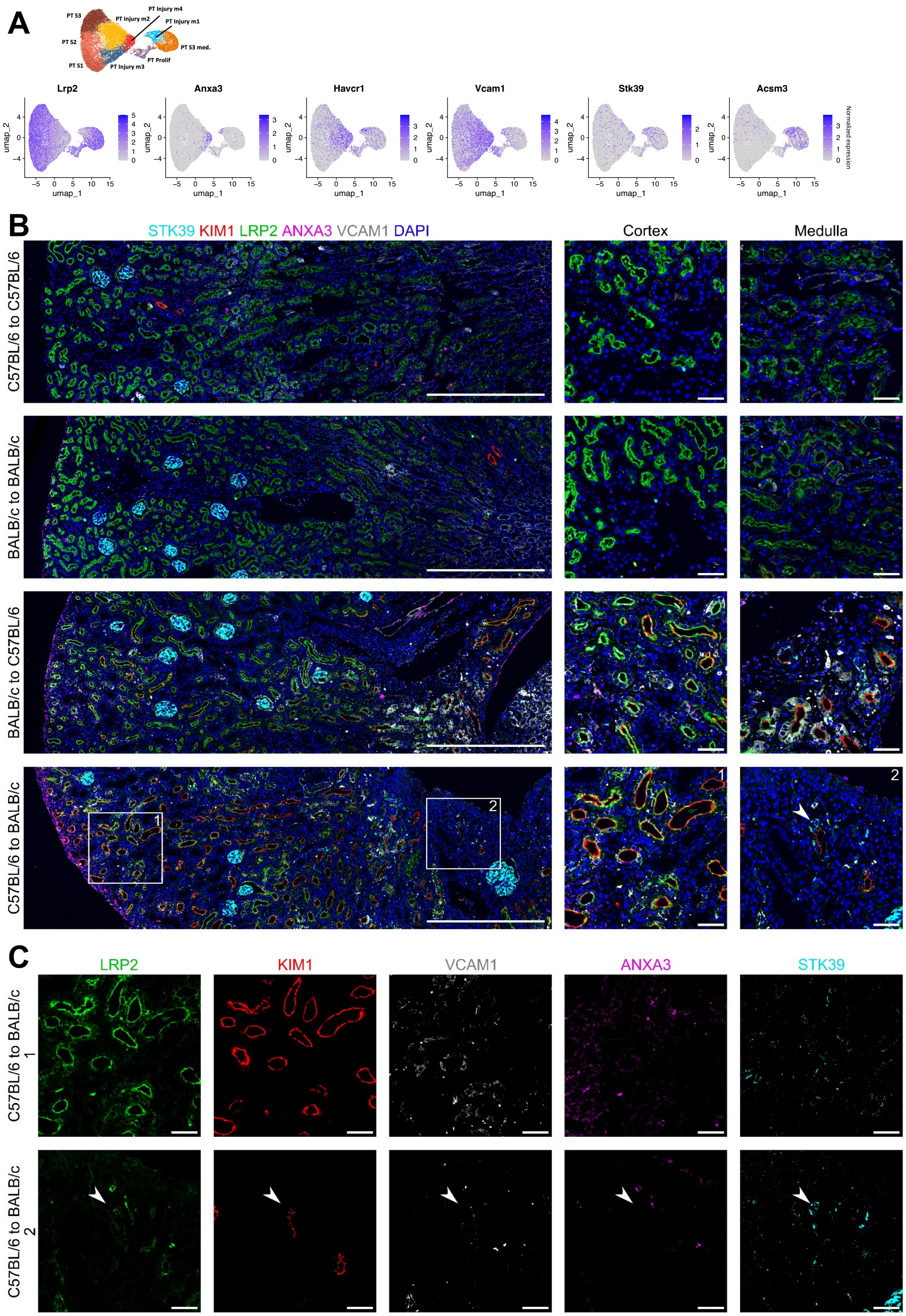

Supplemental Figure S9: Validation of PT Injury m1 and m4. A. *Feature plots of PT cells from mouse snRNA-seq data showing expression of PT Injury m4 marker genes (Anxa3, Havcr1,*

Vcam1) and PT Injury m1 marker genes (Stk39, Acsm3; medullary injury cluster) as well as canonical PT marker gene Lrp2. Expression domains partially overlap, with PT Injury m4 markers also showing expression within the PT Injury m1 domain. ***B.*** *Immunofluorescence staining for STK39, VCAM1, ANXA3 and KIM1 (encoded by Havcr1) in syngeneic and allogeneic mouse kidneys. In allogeneic kidneys, STK39 (cyan) shows distinct medullary proximal tubule expression, often separated from the VCAM1 (white) domain. ANXA3 (magenta) overlaps with KIM1 (red) but not with STK39. All marker signals co-localize with the canonical proximal tubule marker LRP2 (green). Comparative analysis with syngeneic kidneys confirms the specificity and spatial distribution of marker expression. Scale bars: 500 µm (large image); 50 µm (cortex and medulla). All experiments repeated three times with similar result.* ***C.*** *Single-channel images of the stained markers in allogeneic transplanted kidneys (C57BL/6 to BALB/c) show conserved spatial localization and marker co-expression in cortical (1) and medullary (2) regions. Nuclei are counterstained with DAPI. Scale bar: 50 µm.*

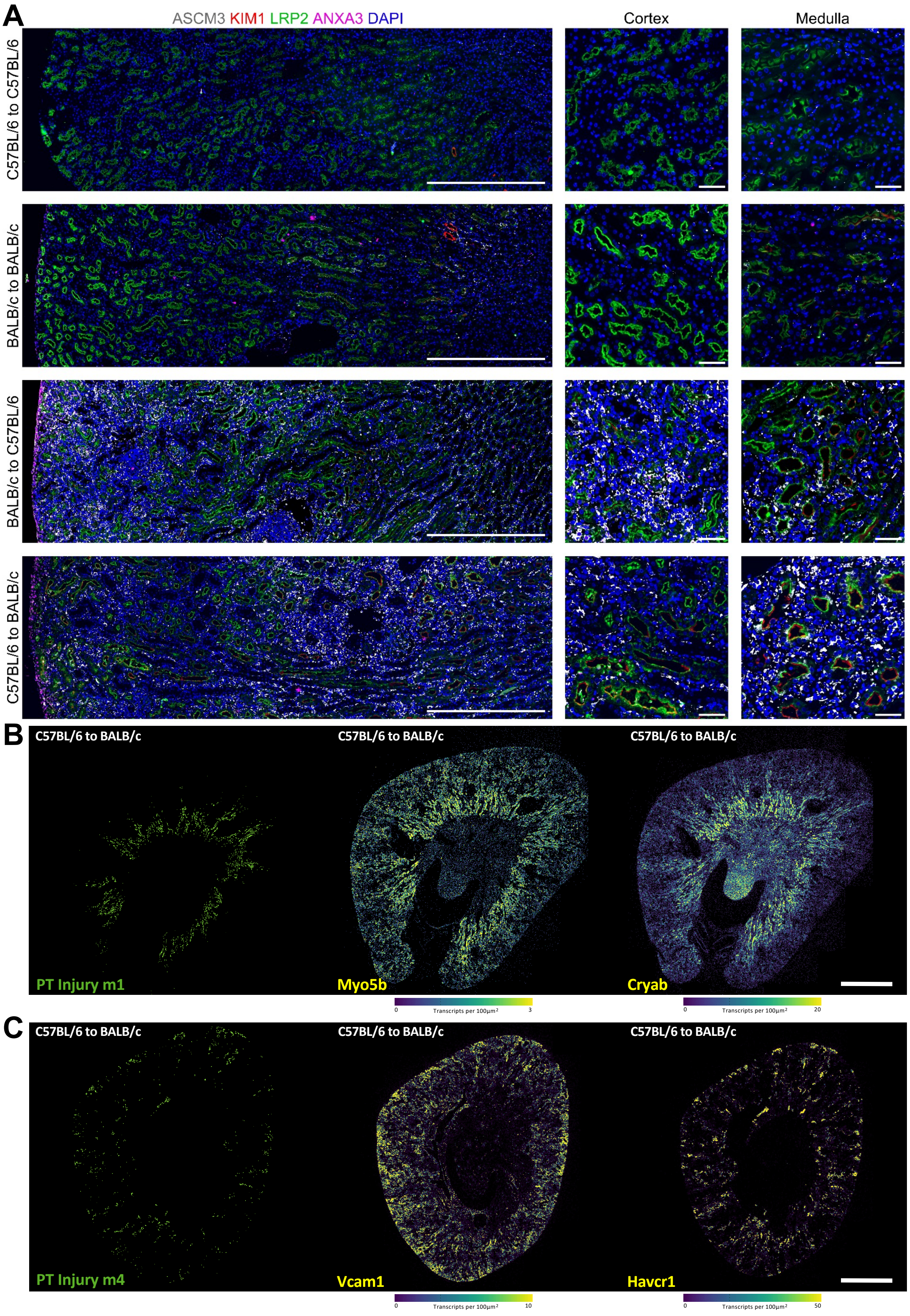
A
ASCM3 KIM1 LRP2 ANXA3 DAPI
Cortex
Medulla
C57BL/6 to C57BL/6
BALB/c to BALB/c
BALB/c to C57BL/6
C57BL/6 to BALB/c
B
C57BL/6 to BALB/c
C57BL/6 to BALB/c
C57BL/6 to BALB/c
PT Injury m1
Myo5b
Cryab
0
Transcripts per 100μm²
3
0
Transcripts per 100μm²
20
C
C57BL/6 to BALB/c
C57BL/6 to BALB/c
C57BL/6 to BALB/c
PT Injury m4
Vcam1
Havcr1
0
Transcripts per 100μm²
10
0
Transcripts per 100μm²
50

Supplemental Figure S10: Validation of PT Injury m1 and m4. A. *Immunofluorescence staining for the PT Injury m1 marker ACSM3 and PT Injury m4 markers ANXA3 and KIM1 (HAVCR1), together with the canonical PT marker LRP2. Expression domains partially overlap, as shown in the feature plots (Supplementary Fig. S9A). PT Injury m4 markers also display some expression within the PT Injury m1 domain. In allogeneic mouse kidneys, ACSM3 (white) shows distinct expression in medullary proximal tubules, demonstrated by co-localization with LRP2 (green). ACSM3-positive cells are predominantly positive for KIM1 (red) but negative for ANXA3 (magenta). Nuclei are counterstained with DAPI. Scale bars: 500 µm (large image); 50 µm (cortex and medulla). All experiments repeated three times with similar result.* ***B.*** *Gene density plots for PT Injury m1 marker genes Myo5b and Cryab, and* ***C.*** *for PT Injury m4 marker genes Vcam1 and Havcr1 from spatial transcriptomics data. Scale bar: 1000 µm. Transcript densities are represented according to the color scale. Left panels depict the spatial localization of PT Injury m1 and m4 cell populations as inferred from snRNA-seq label transfer, respectively. All experiments repeated for all allogeneic spatial transcriptomic samples with similar result.*

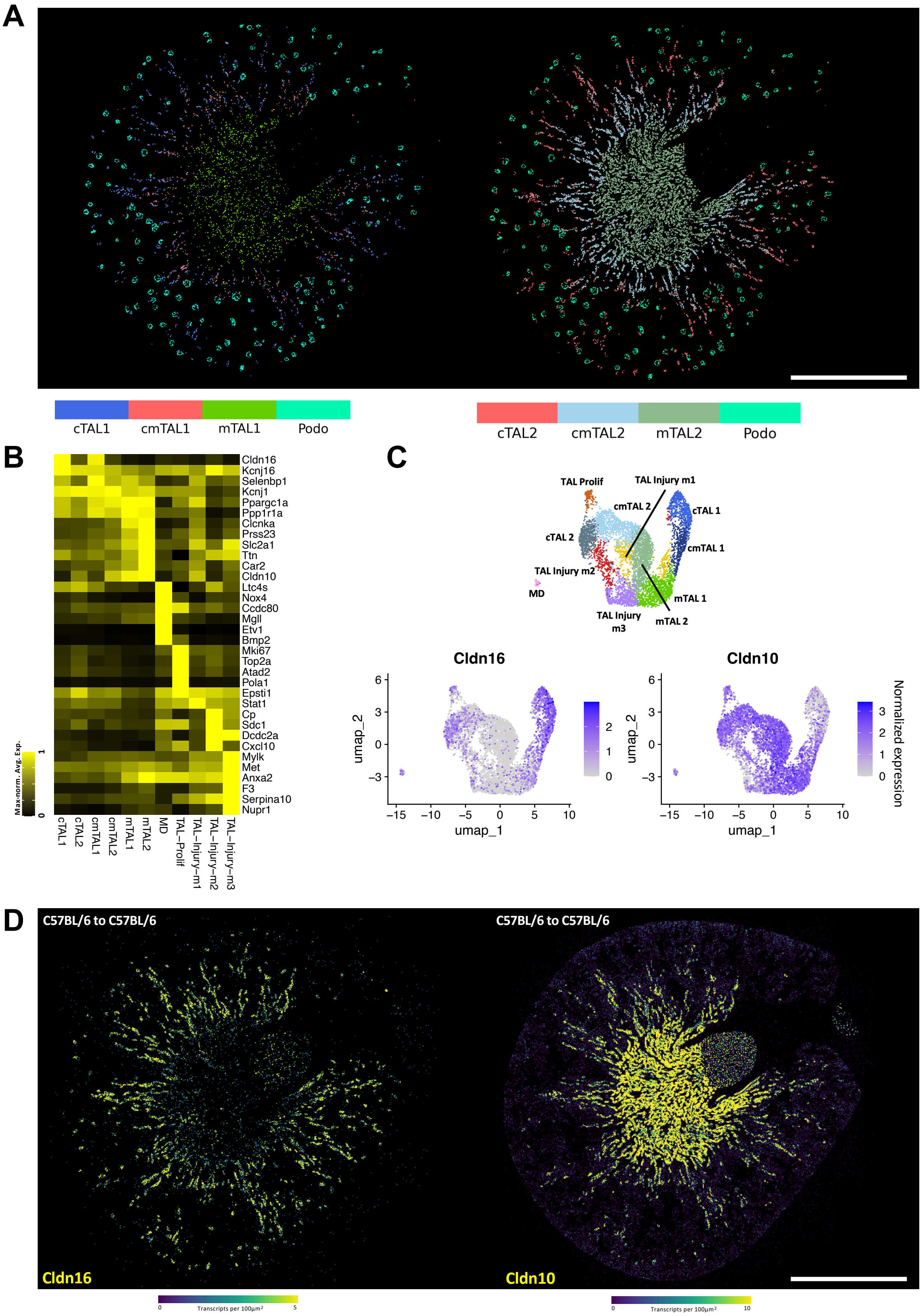

A
cTAL1
cmTAL1
mTAL1
Podo
cTAL2
cmTAL2
mTAL2
Podo
B
Cldn16
Kcnj16
Selenbp1
Kcnj1
Ppargc1a
Ppp1r1a
Clcnka
Prss23
Slc2a1
Ttn
Car2
Cldn10
Ltc4s
Nox4
Ccdc80
Mgll
Etv1
Bmp2
Mki67
Top2a
Atad2
Pola1
Epsti1
Stat1
Cp
Sdc1
Dcdc2a
Cxcl10
Mylk
Met
Anxa2
F3
Serpina10
Nupr1
Max-norm. Avg. Exp.
cTAL1
cTAL2
cmTAL1
cmTAL2
mTAL1
mTAL2
MD
TAL−Prolif
TAL−Injury−m1
TAL−Injury−m2
TAL−Injury−m3
C
TAL Prolif
TAL Injury m1
cmTAL 2
cTAL 1
cTAL 2
cmTAL 1
TAL Injury m2
MD
mTAL 1
TAL Injury m3
mTAL 2
Cldn16
Cldn10
umap_2
umap_1
Normalized expression
D
C57BL/6 to C57BL/6
C57BL/6 to C57BL/6
Cldn16
Cldn10
Transcripts per 100μm²
Transcripts per 100μm²

Supplemental Figure S11: Spatial distribution of healthy TAL segments from snRNA-seq label transfer. A. *Distribution of TAL subtypes obtained by SingleR-based label transfer from snRNA-seq data. Podocytes are included to facilitate identification of the kidney cortex. cTAL1 and cmTAL1 show elevated Cldn16 expression (with very residual expression in mTAL1) while cTAL2, cmTAL2 and mTAL2 are predominantly expressing Cldn10. All experiments repeated for all spatial transcriptomic samples with similar result.* ***B.*** *Expression of snRNA-seq-derived TAL marker genes in spatial transcriptomics data (see Fig. 3 for expression in snRNA-seq data).* ***C.*** *Feature plots of the mentioned claudins, Cldn10 and Cldn16 in TAL cells from snRNA-seq data.* ***D.*** *Transcript density plots for Cldn16 and Cldn10 reflecting the mentioned cTAL1-cmTAL1-mTAL1 and cTAL2-cmTAL2-mTAL2 axes. Transcript densities are represented according to the color scale. Scale bar: 1000 µm. Source data are provided as a Source Data file. All experiments repeated for all spatial transcriptomic samples with similar result.*

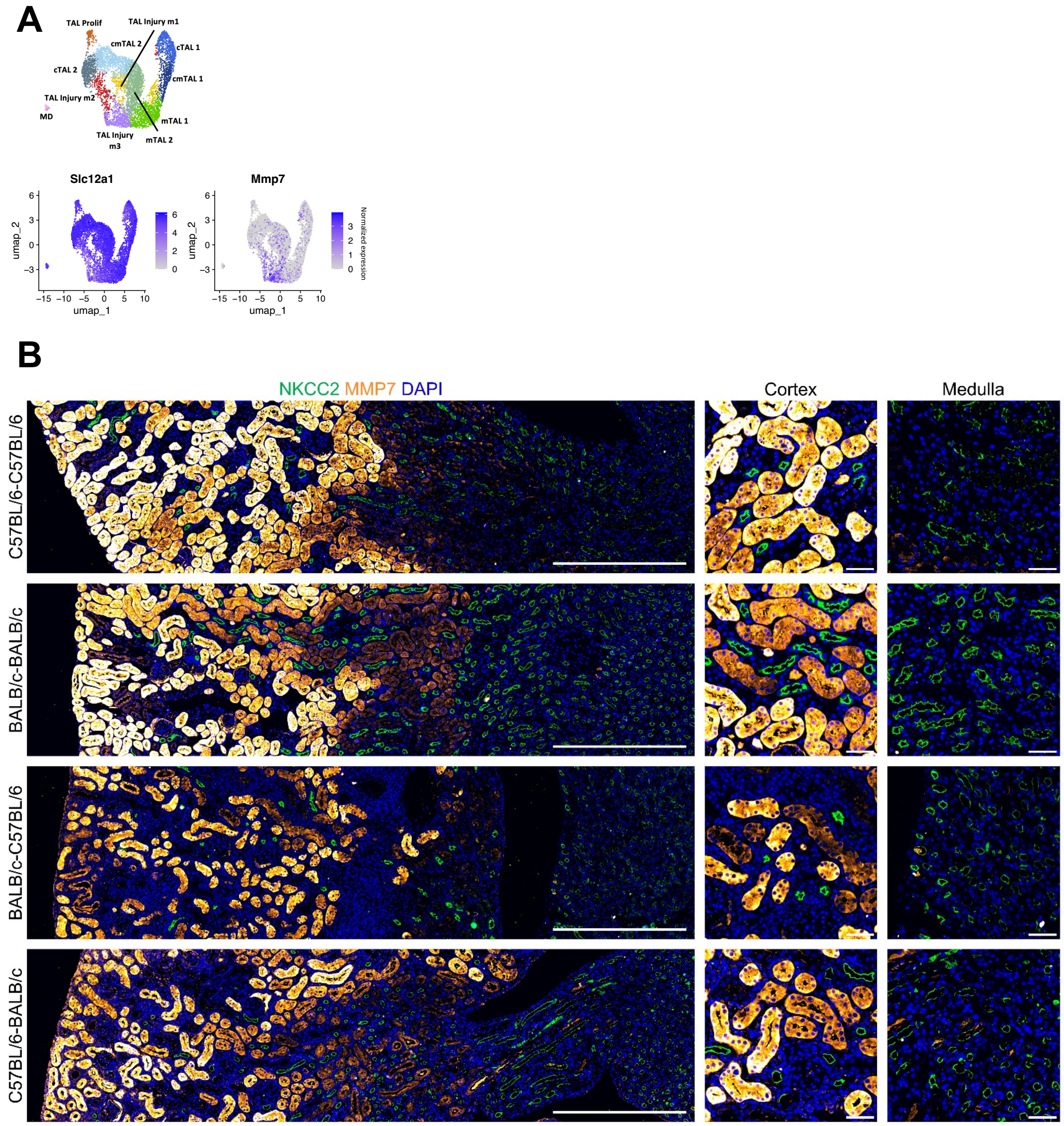

Supplemental Figure S12: Validation of TAL Injury m3. A. *Feature plots of TAL cells from mouse snRNA-seq data showing expression of TAL Injury m3 marker gene Mmp7 as well as canonical TAL marker gene Slc12a1.* ***B.*** *Immunofluorescence staining for MMP7. Within the TAL, the TAL Injury m3 marker MMP7 is expressed in scattered medullary NKCC2-positive TAL cells in allogeneic transplanted kidneys but is absent in syngeneic grafts (Slc12a1 encodes NKCC2). The predominant medullary localization of TAL Injury m3 was anticipated from label transfer of snRNA-seq data to the spatial transcriptomics dataset (see Fig. 3G). Nuclei are counterstained with DAPI. MMP7 also labels PT cells. The lower panels, also shown in Fig. 3J, are reproduced here for convenience. Scale bars: 500 µm (large image); 50 µm (cortex and medulla). All experiments repeated three times with similar result.*

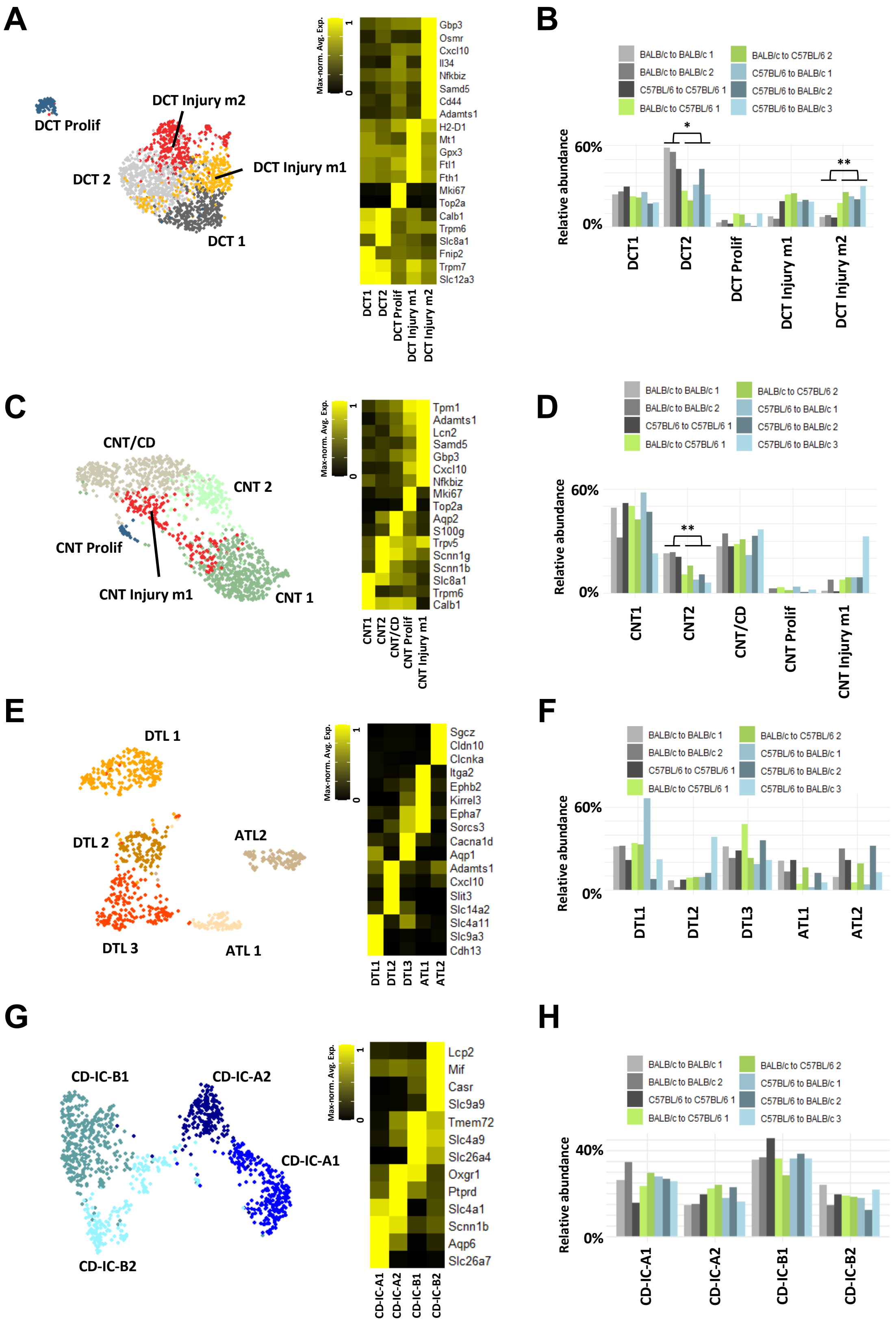

A
DCT Prolif
DCT Injury m2
DCT 2
DCT Injury m1
DCT 1
Max-norm. Avg. Exp.
1
0
Gbp3
Osmr
Cxcl10
Il34
Nfkbiz
Samd5
Cd44
Adamts1
H2-D1
Mt1
Gpx3
Ftl1
Fth1
Mki67
Top2a
Calb1
Trpm6
Slc8a1
Fnip2
Trpm7
Slc12a3
DCT1
DCT2
DCT Prolif
DCT Injury m1
DCT Injury m2
B
BALB/c to BALB/c 1
BALB/c to BALB/c 2
C57BL/6 to C57BL/6 1
BALB/c to C57BL/6 1
BALB/c to C57BL/6 2
C57BL/6 to BALB/c 1
C57BL/6 to BALB/c 2
C57BL/6 to BALB/c 3
Relative abundance
60%
0%
*
**
DCT1
DCT2
DCT Prolif
DCT Injury m1
DCT Injury m2
C
CNT/CD
CNT 2
CNT Prolif
CNT Injury m1
CNT 1
Max-norm. Avg. Exp.
1
0
Tpm1
Adamts1
Lcn2
Samd5
Gbp3
Cxcl10
Nfkbiz
Mki67
Top2a
Aqp2
S100g
Trpv5
Scnn1g
Scnn1b
Slc8a1
Trpm6
Calb1
CNT1
CNT2
CNT/CD
CNT Prolif
CNT Injury m1
D
BALB/c to BALB/c 1
BALB/c to BALB/c 2
C57BL/6 to C57BL/6 1
BALB/c to C57BL/6 1
BALB/c to C57BL/6 2
C57BL/6 to BALB/c 1
C57BL/6 to BALB/c 2
C57BL/6 to BALB/c 3
Relative abundance
60%
0%
**
CNT1
CNT2
CNT/CD
CNT Prolif
CNT Injury m1
E
DTL 1
DTL 2
ATL2
DTL 3
ATL 1
Max-norm. Avg. Exp.
1
0
Sgcz
Cldn10
Clcnka
Itga2
Ephb2
Kirrel3
Epha7
Sorcs3
Cacna1d
Aqp1
Adamts1
Cxcl10
Slit3
Slc14a2
Slc4a11
Slc9a3
Cdh13
DTL1
DTL2
DTL3
ATL1
ATL2
F
BALB/c to BALB/c 1
BALB/c to BALB/c 2
C57BL/6 to C57BL/6 1
BALB/c to C57BL/6 1
BALB/c to C57BL/6 2
C57BL/6 to BALB/c 1
C57BL/6 to BALB/c 2
C57BL/6 to BALB/c 3
Relative abundance
60%
0%
DTL1
DTL2
DTL3
ATL1
ATL2
G
CD-IC-B1
CD-IC-A2
CD-IC-A1
CD-IC-B2
Max-norm. Avg. Exp.
1
0
Lcp2
Mif
Casr
Slc9a9
Tmem72
Slc4a9
Slc26a4
Oxgr1
Ptprd
Slc4a1
Scnn1b
Aqp6
Slc26a7
CD-IC-A1
CD-IC-A2
CD-IC-B1
CD-IC-B2
H
BALB/c to BALB/c 1
BALB/c to BALB/c 2
C57BL/6 to C57BL/6 1
BALB/c to C57BL/6 1
BALB/c to C57BL/6 2
C57BL/6 to BALB/c 1
C57BL/6 to BALB/c 2
C57BL/6 to BALB/c 3
Relative abundance
40%
0%
CD-IC-A1
CD-IC-A2
CD-IC-B1
CD-IC-B2

Supplemental Figure S13: Results from subclustering analysis of remaining major cell types. *Note that subclustering of leukocytes is included in the main figures. Analyses as indicated for DCT (**A.** and **B.**), CNT (**C.** and **D.**), TL (**E.** and **F.,** ATL – ascending thin limb, DTL – descending thin limb) and CD-IC **(G.** and **H.**). Heatmaps show marker gene expression (counts per million, maximum-normalized per gene). Student's t-test with Benjamini-Hochberg correction for multiple testing. P-value: *<0.05, **<0.005, no asterisk – not significant. Source data are provided as a Source Data file.*

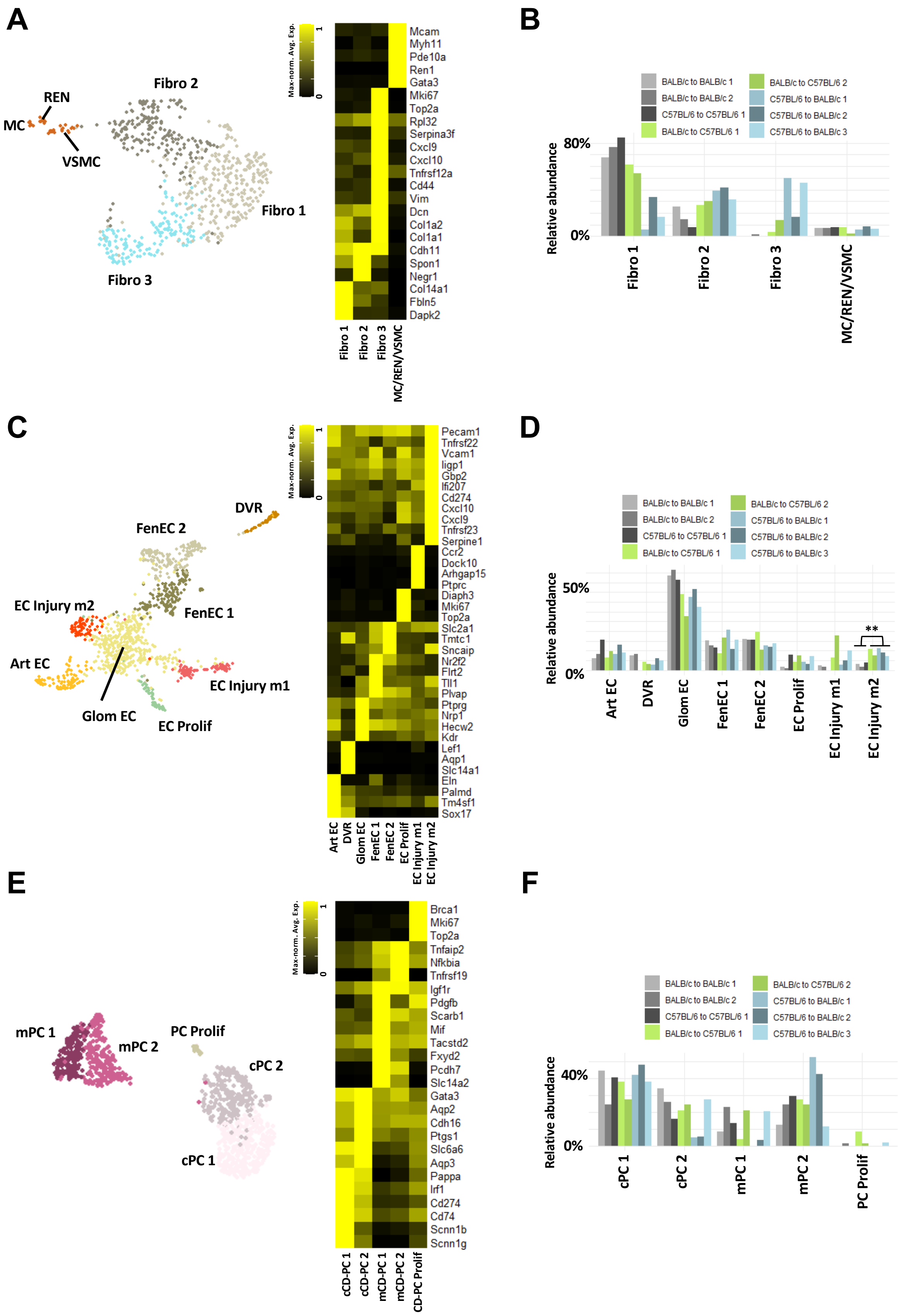
A
REN
MC
VSMC
Fibro 2
Fibro 1
Fibro 3
Max-norm. Avg. Exp.
1
0
Mcam
Myh11
Pde10a
Ren1
Gata3
Mki67
Top2a
Rpl32
Serpina3f
Cxcl9
Cxcl10
Tnfrsf12a
Cd44
Vim
Dcn
Col1a2
Col1a1
Cdh11
Spon1
Negr1
Col14a1
Fbln5
Dapk2
Fibro 1
Fibro 2
Fibro 3
MC/REN/VSMC
B
BALB/c to BALB/c 1
BALB/c to BALB/c 2
C57BL/6 to C57BL/6 1
BALB/c to C57BL/6 1
BALB/c to C57BL/6 2
C57BL/6 to BALB/c 1
C57BL/6 to BALB/c 2
C57BL/6 to BALB/c 3
Relative abundance
80%
0%
Fibro 1
Fibro 2
Fibro 3
MC/REN/VSMC
C
DVR
FenEC 2
FenEC 1
EC Injury m2
Art EC
Glom EC
EC Prolif
EC Injury m1
Max-norm. Avg. Exp.
1
0
Pecam1
Tnfrsf22
Vcam1
Iigp1
Gbp2
Ifi207
Cd274
Cxcl10
Cxcl9
Tnfrsf23
Serpine1
Ccr2
Dock10
Arhgap15
Ptprc
Diaph3
Mki67
Top2a
Slc2a1
Tmtc1
Sncaip
Nr2f2
Flrt2
Tll1
Plvap
Ptprg
Nrp1
Hecw2
Kdr
Lef1
Aqp1
Slc14a1
Eln
Palmd
Tm4sf1
Sox17
Art EC
DVR
Glom EC
FenEC 1
FenEC 2
EC Prolif
EC Injury m1
EC Injury m2
D
BALB/c to BALB/c 1
BALB/c to BALB/c 2
C57BL/6 to C57BL/6 1
BALB/c to C57BL/6 1
BALB/c to C57BL/6 2
C57BL/6 to BALB/c 1
C57BL/6 to BALB/c 2
C57BL/6 to BALB/c 3
Relative abundance
50%
0%
**
Art EC
DVR
Glom EC
FenEC 1
FenEC 2
EC Prolif
EC Injury m1
EC Injury m2
E
mPC 1
mPC 2
PC Prolif
cPC 2
cPC 1
Max-norm. Avg. Exp.
1
0
Brca1
Mki67
Top2a
Tnfaip2
Nfkbia
Tnfrsf19
Igf1r
Pdgfb
Scarb1
Mif
Tacstd2
Fxyd2
Pcdh7
Slc14a2
Gata3
Aqp2
Cdh16
Ptgs1
Slc6a6
Aqp3
Pappa
Irf1
Cd274
Cd74
Scnn1b
Scnn1g
cCD-PC 1
cCD-PC 2
mCD-PC 1
mCD-PC 2
CD-PC Prolif
F
BALB/c to BALB/c 1
BALB/c to BALB/c 2
C57BL/6 to C57BL/6 1
BALB/c to C57BL/6 1
BALB/c to C57BL/6 2
C57BL/6 to BALB/c 1
C57BL/6 to BALB/c 2
C57BL/6 to BALB/c 3
Relative abundance
40%
0%
cPC 1
cPC 2
mPC 1
mPC 2
PC Prolif

Supplemental Figure S14: Results from subclustering analysis of remaining major cell types. *Note that subclustering leukocytes in included in the main figures. Analyses as indicated for interstitial cells (**A.** and **B.**, MC – mesangial cells, REN – renin-producing cells, VSMC – vascular smooth muscle cells, Fibro - fibroblasts), EC (**C.** and **D.**, FenEC – fenestrated EC, Glom EC – glomerular EC, Art EC – arterial EC, DVR – descending vasa recta) and CD-PC (**E.** and **F.,** mPC – medullary principal cells, cPC – cortical principal cells). Heatmaps show marker gene expression (counts per million, maximum-normalized per gene). Student's t-test with Benjamini-Hochberg correction for multiple testing. P-value: **<0.005, no asterisk – not significant. Source data are provided as a Source Data file.*

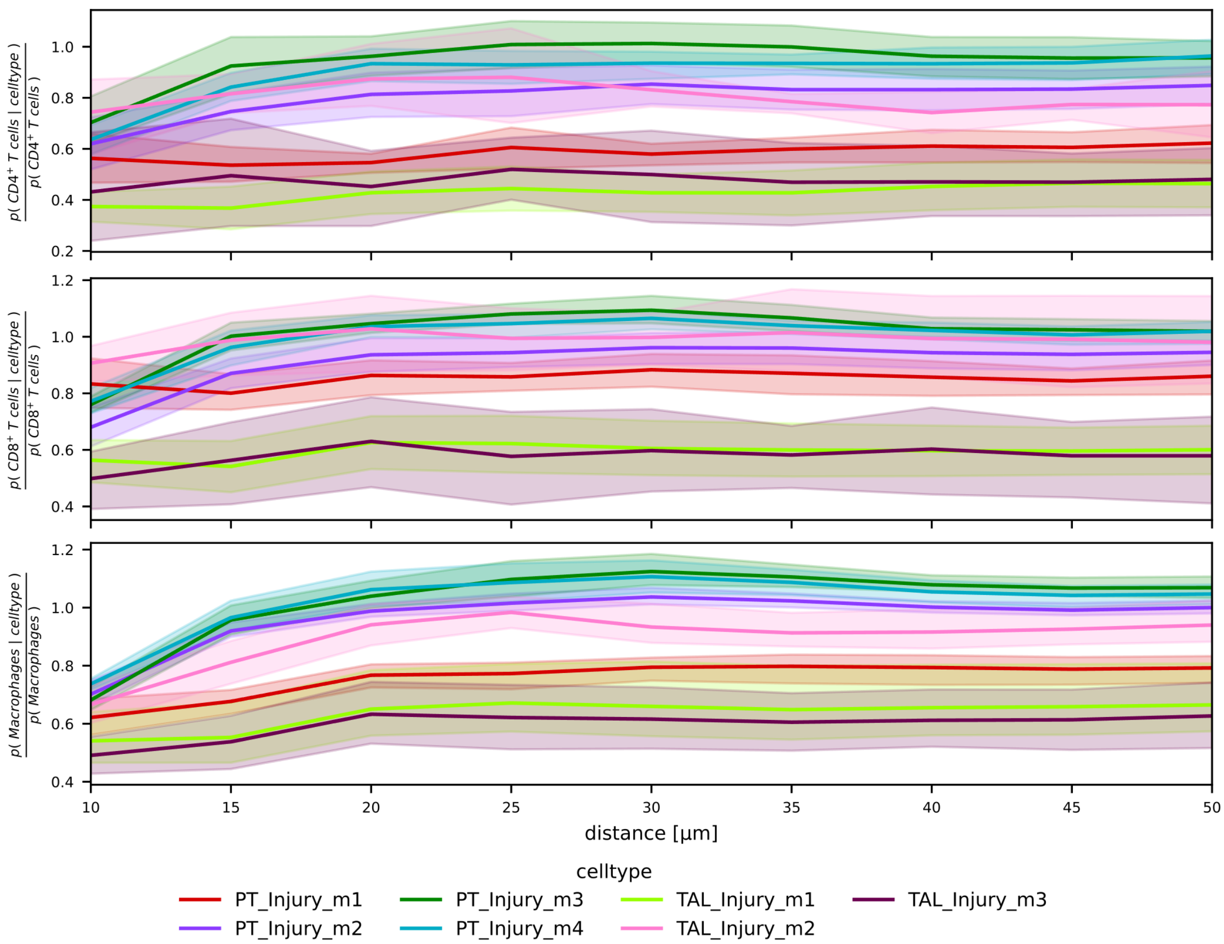

Supplemental Figure S15: Spatial proximity of PT and TAL injury clusters to major immune cell types. *For every injury cluster of interest (e.g. PT Injury m4), the co-occurence to each leukocyte cell type was calculated with squidpy. Briefly, squidpy.gr.co_occurrence was run with a distance interval of 10 to 50 µm with step size 5 based on the provided centroid information from the Xenium data. Compare to analysis for 25 µm in Fig. 4E. Source data are provided as a Source Data file.*

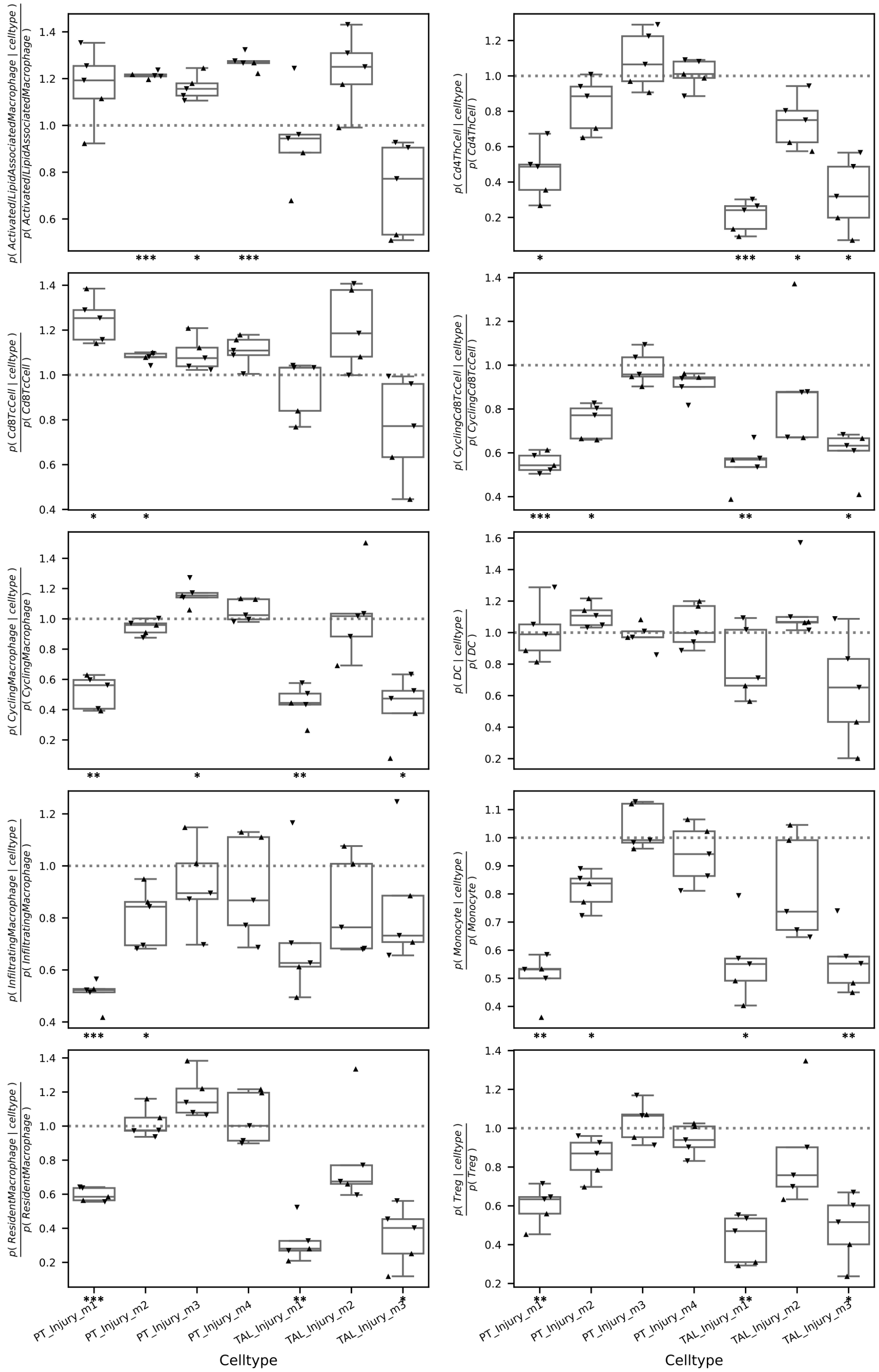

Supplemental Figure S16: Co-occurrence of PT and TAL injury clusters with immune cell cell subtypes. *As in Fig. 4E, we chose 25 µm as indicative of being a direct neighbour.*

Significances from statistical testing below individual boxes. Two-sided one sample t-test with Benjamini-Hochberg correction for multiple testing. P-value: *<0.05, **<0.005, ***<0.001. Source data are provided as a Source Data file. Box plots show the median and the 25th–75th percentiles, with whiskers extending to the most extreme data points within 1.5× the interquartile range. Analogous to Fig. 4E, allogeneic transplantation C57BL/6 to BALB/c (triangles pointing downwards, $n = 3$) and BALB/c to C57BL/6 (triangles pointing upwards, $n = 2$).

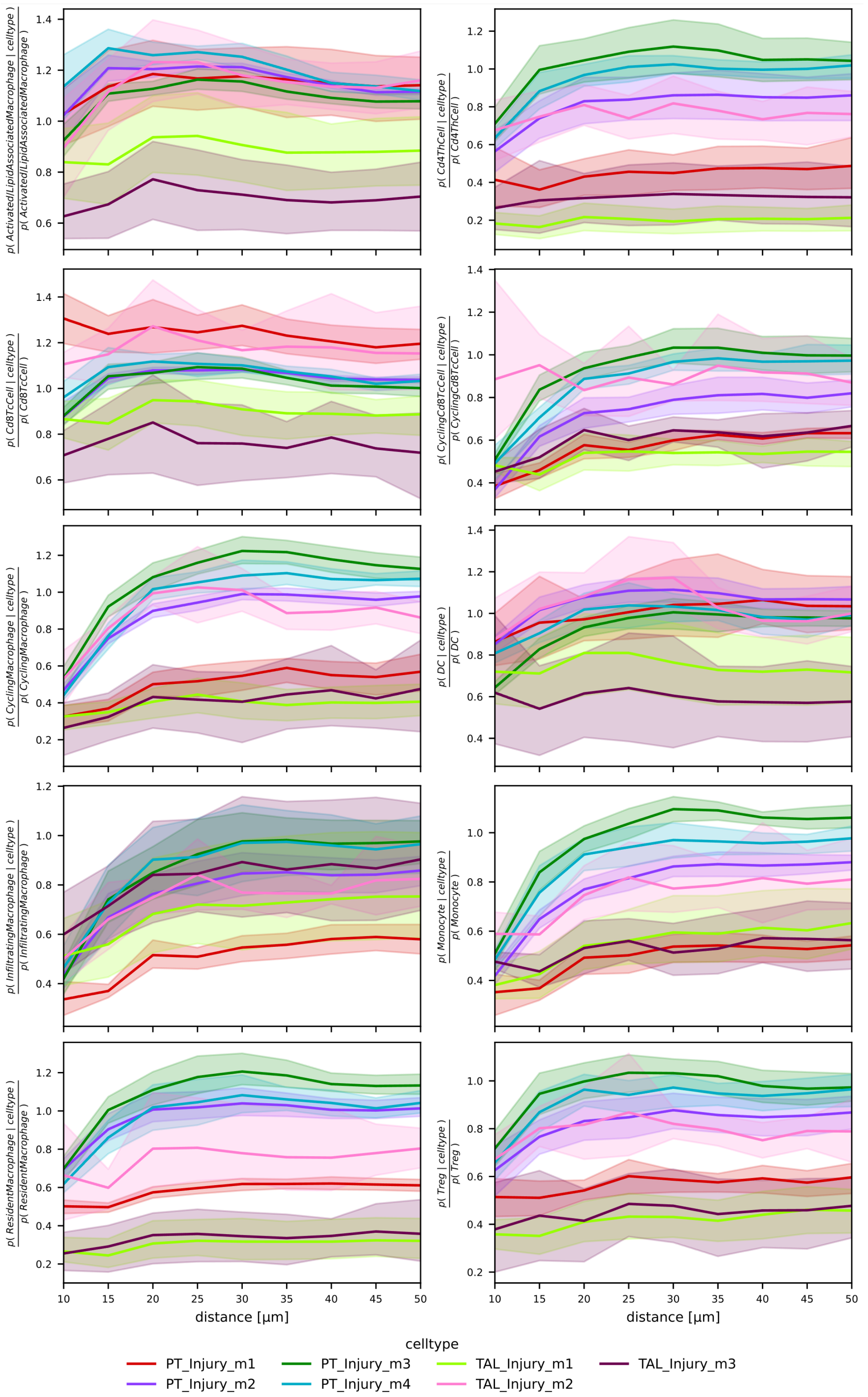
p(Activated/LipidAssociatedMacrophage | celltype) / p(Activated/LipidAssociatedMacrophage)
p(Cd4ThCell | celltype) / p(Cd4ThCell)
p(Cd8TcCell | celltype) / p(Cd8TcCell)
p(CyclingCd8TcCell | celltype) / p(CyclingCd8TcCell)
p(CyclingMacrophage | celltype) / p(CyclingMacrophage)
p(DC | celltype) / p(DC)
p(InfiltratingMacrophage | celltype) / p(InfiltratingMacrophage)
p(Monocyte | celltype) / p(Monocyte)
p(ResidentMacrophage | celltype) / p(ResidentMacrophage)
p(Treg | celltype) / p(Treg)
distance [μm]
distance [μm]
celltype
PT_Injury_m1
PT_Injury_m2
PT_Injury_m3
PT_Injury_m4
TAL_Injury_m1
TAL_Injury_m2
TAL_Injury_m3

Supplemental Figure S17: Spatial proximity of PT and TAL injury clusters to immune cell subtypes. *Figure analogous to Suppl. Fig. S15 for immune cell subtypes. Source data are provided as a Source Data file.*

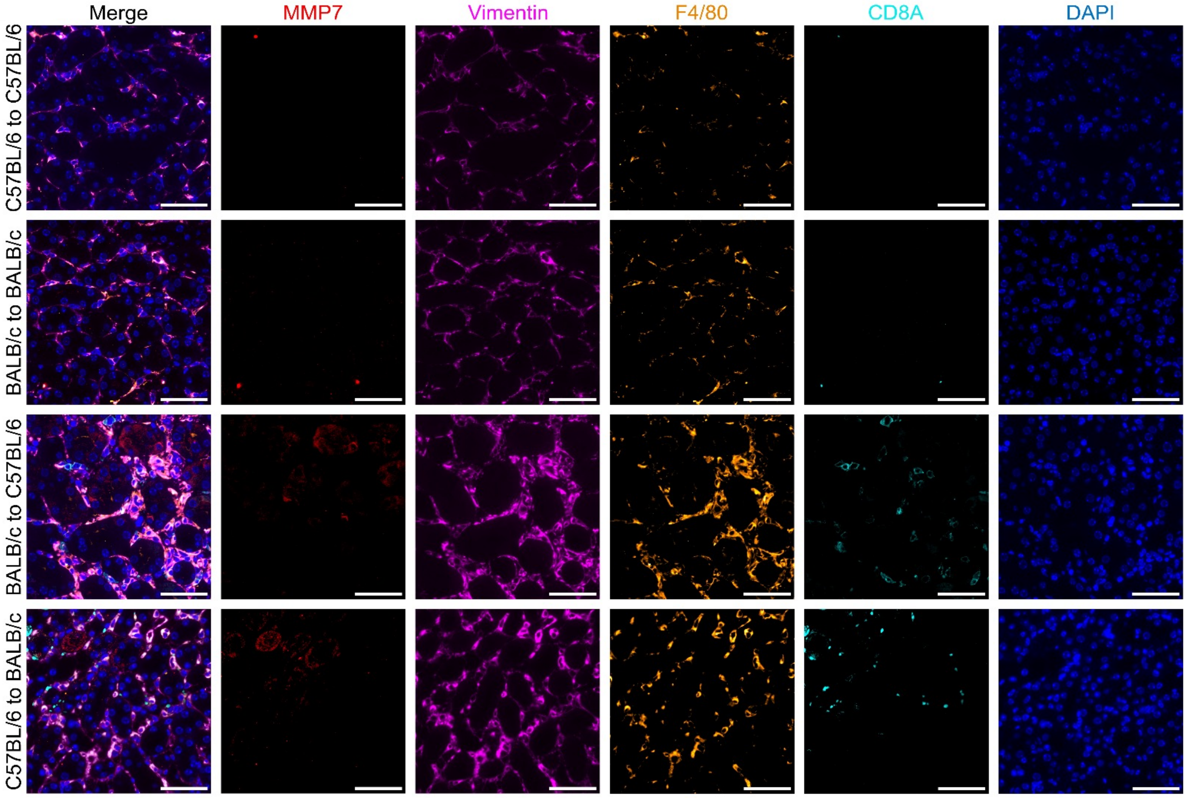

Supplemental Figure S18: Localization of MMP7-positive TAL cells (TAL Injury m3) with fibroblasts, macrophages and CD8A-positive T cells. *Immunofluorescence staining for F4/80 (macrophages, orange), CD8A ($CD8^+$ T cells, cyan), Vimentin (Vim, fibroblasts and macrophages, magenta) and MMP7 (TAL Injury m3, red). Injured TAL cells show markedly fewer or no direct contacts with immune cells compared to PT Injury m4 (see Fig. 5E). Nuclei are counterstained with DAPI. Scale bar: 50 µm. All experiments repeated three times with similar result.*

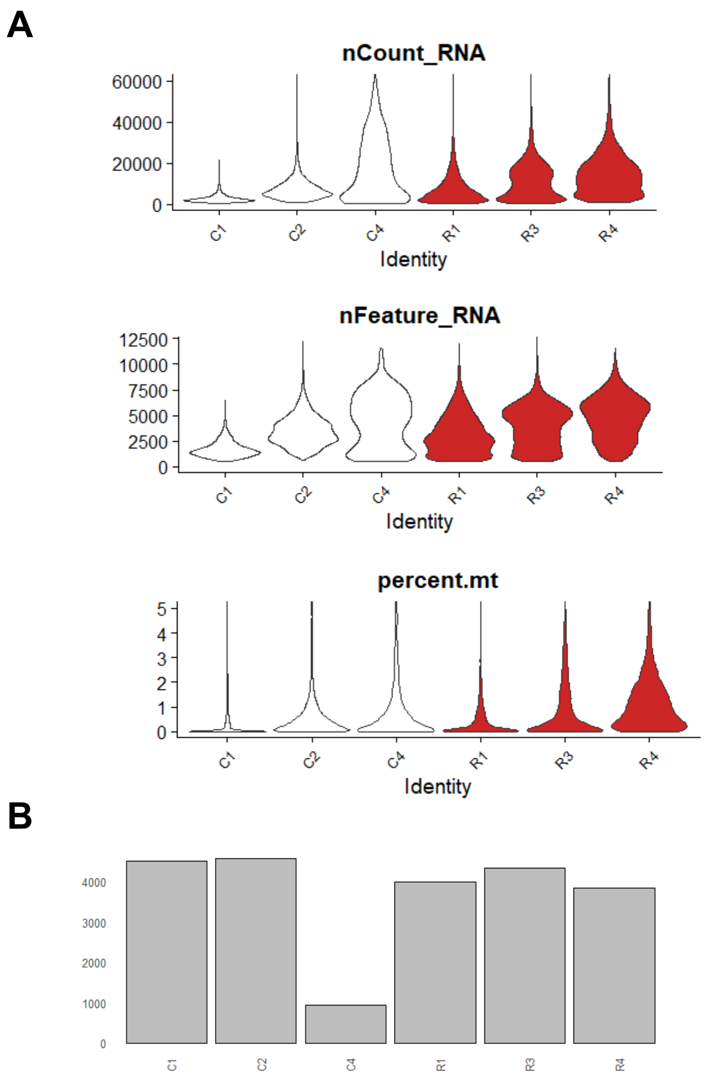

Supplemental Figure S19: Quality metrics for human snRNA-seq data. A. *Number of detected genes (nFeature_RNA), transcripts (nCount_RNA) and percentage of mitochondrial reads (percent.mt) per sample.* ***B.*** *Number of detected cells per sample. Source data are provided as a Source Data file.*

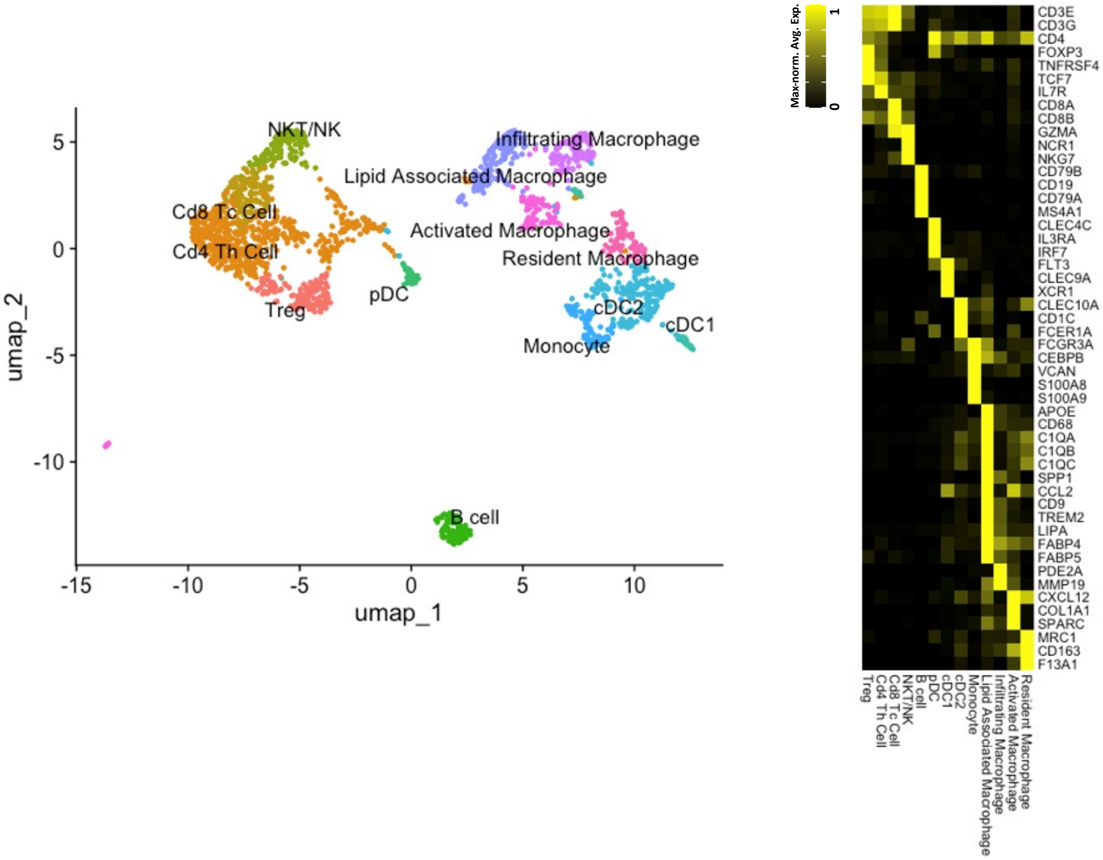

Supplemental Figure S20: Subclustering of human leukocytes. *UMAP of human snRNA-seq leukocyte subclustering with marker gene heatmap on the right side (maximum-normalized gene expression). Source data are provided as a Source Data file.*

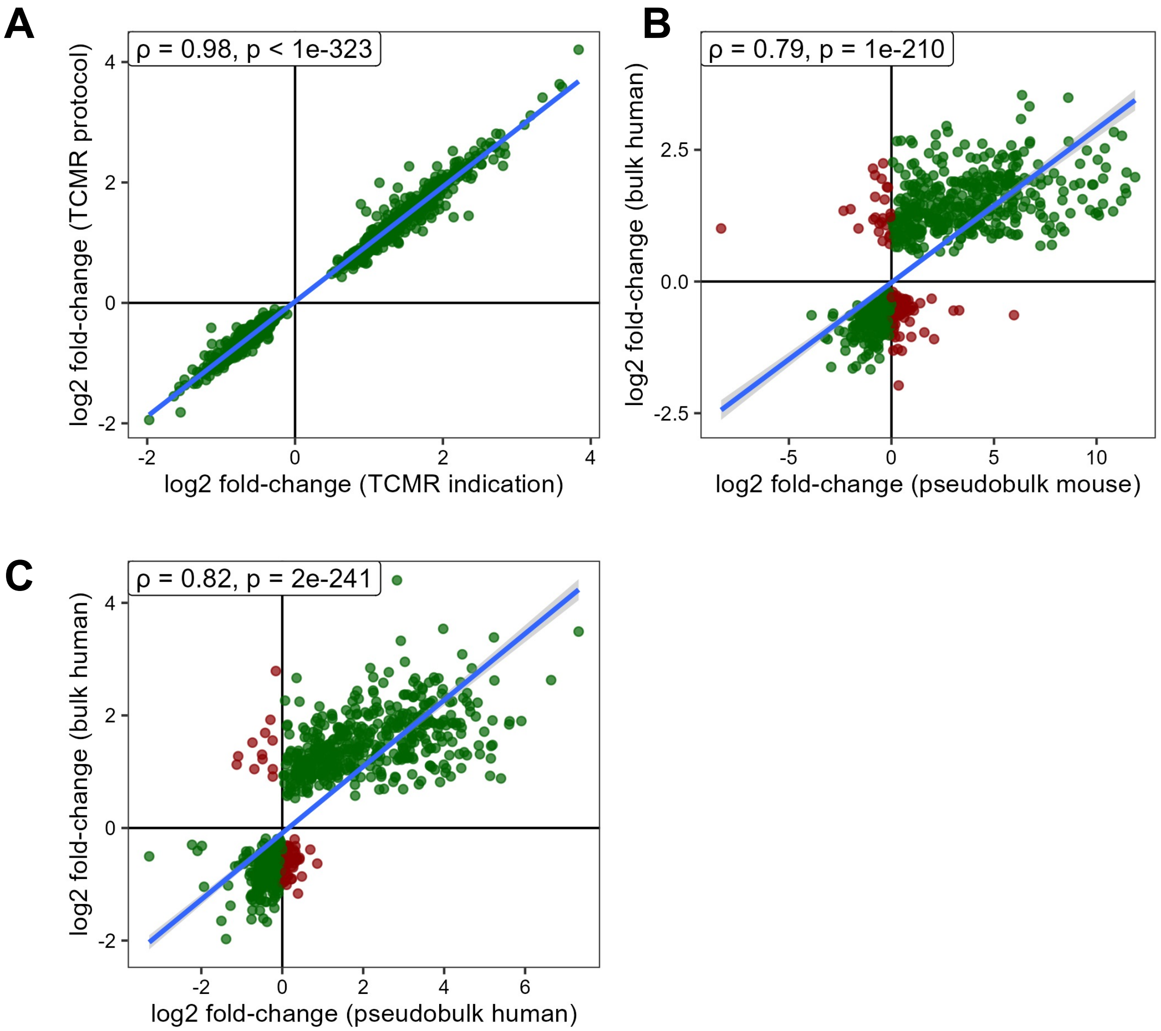

Supplemental Figure S21: Gene expression profiles of TCMR are conserved across single nuclei and bulk transcriptomics data. A. *Bulk microarray log2 fold-changes in the top 500 increased and decreased genes in TCMR versus no rejection (NR) were similar among protocol and indication biopsies (N TCMR protocol = 22, N TCMR indication = 140).* ***B.*** *Pseudobulk log2 fold-changes in the top 500 increased and decreased genes in TCMR versus NR (from bulk transcriptomics) with fold changes from mouse snRNA-seq pseudobulk on all cells (allogeneic versus syngeneic) were highly correlated.* ***C.*** *Pseudobulk log2 fold-changes in the top 500 increased and decreased genes in TCMR versus NR (from bulk transcriptomics) with fold changes from human snRNA-seq pseudobulk on all cells (TCMR versus stable allograft) were highly correlated. Green circles denote individual genes that were differentially expressed in the same direction between assays. Red circles denote individual genes that were differentially expressed in the opposite direction between assays. Solid blue line denotes the fitted regression*

in log2 fold changes with 95% confidence intervals. ρ denotes the spearman correlation coefficients with respective p-value. Source data are provided as a Source Data file.

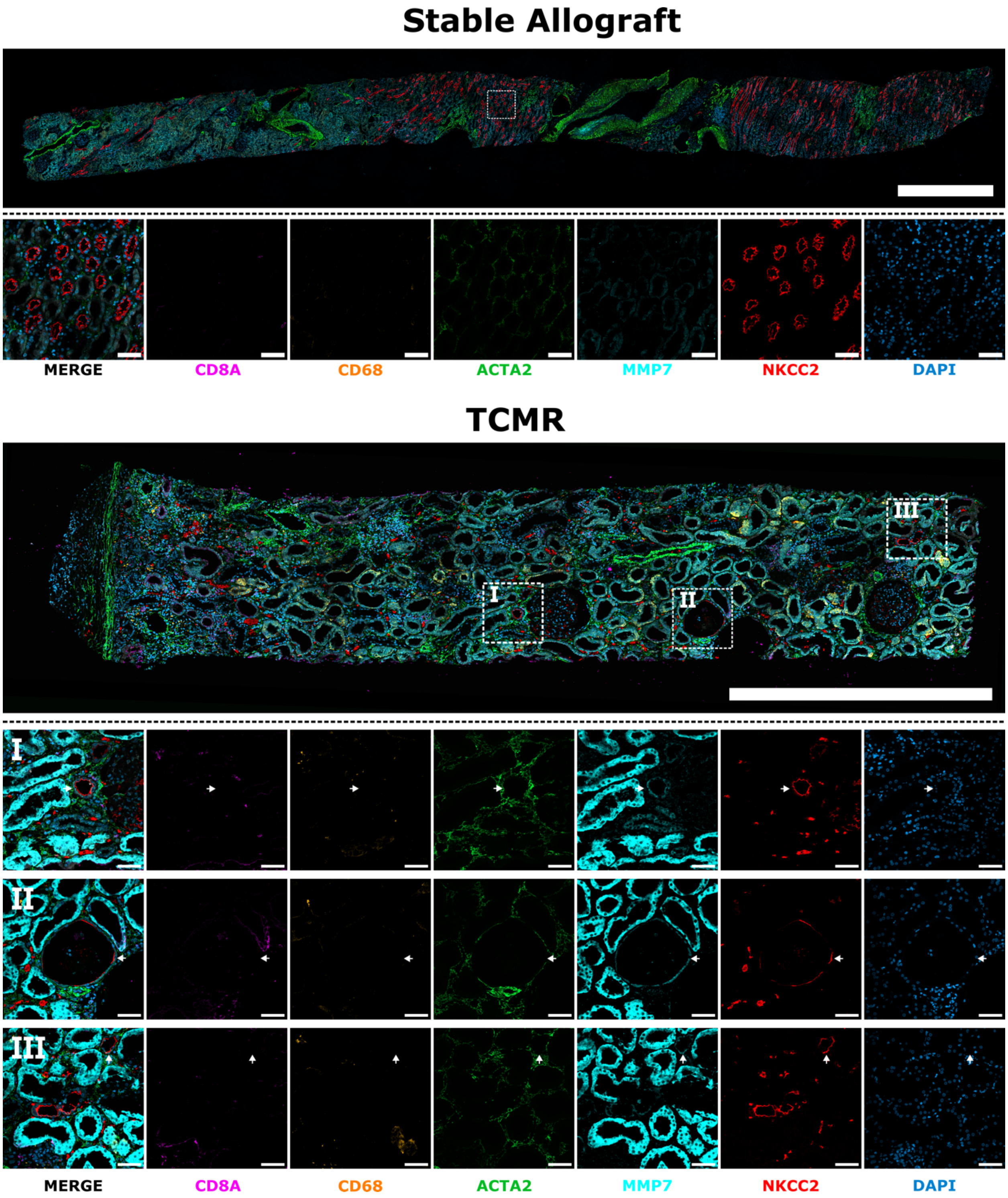

Supplemental Figure S22: Representative overview and inset images of microenvironments surrounding injured TAL cells in kidney biopsy sections from patients with stable allograft function and patients with TCMR. *Immunofluorescence staining for T-cells (CD8A, magenta), macrophages (CD68, yellow) and fibroblasts (ACTA2, alpha-smooth*

muscle actin, green). Injured TAL cells (TAL Injury h2) (arrowheads) are identified by expression of NKCC2 (red) and MMP7 (cyan). Nuclei are stained with DAPI (blue). Scale bars of overview images: 1000µm. Scale bars of insets: 50µm. All experiments repeated three times with similar result.

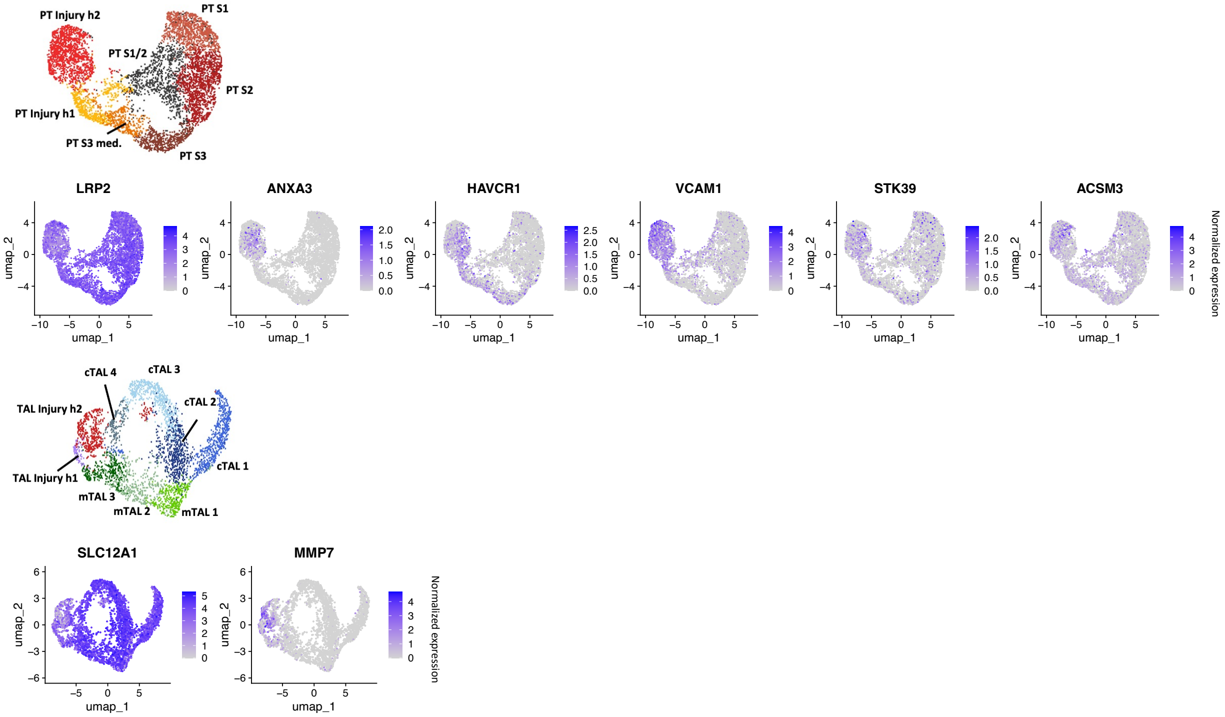

Supplemental Figure S23: Expression domains of selected PT and TAL marker genes. *Marker genes include all genes used in immunofluorescence validation for PT Injury h2 and TAL Injury h2 as well as canonical markers LRP2 for PT and SLC12A1 for TAL (Fig. 7, Suppl. Fig. S22).*

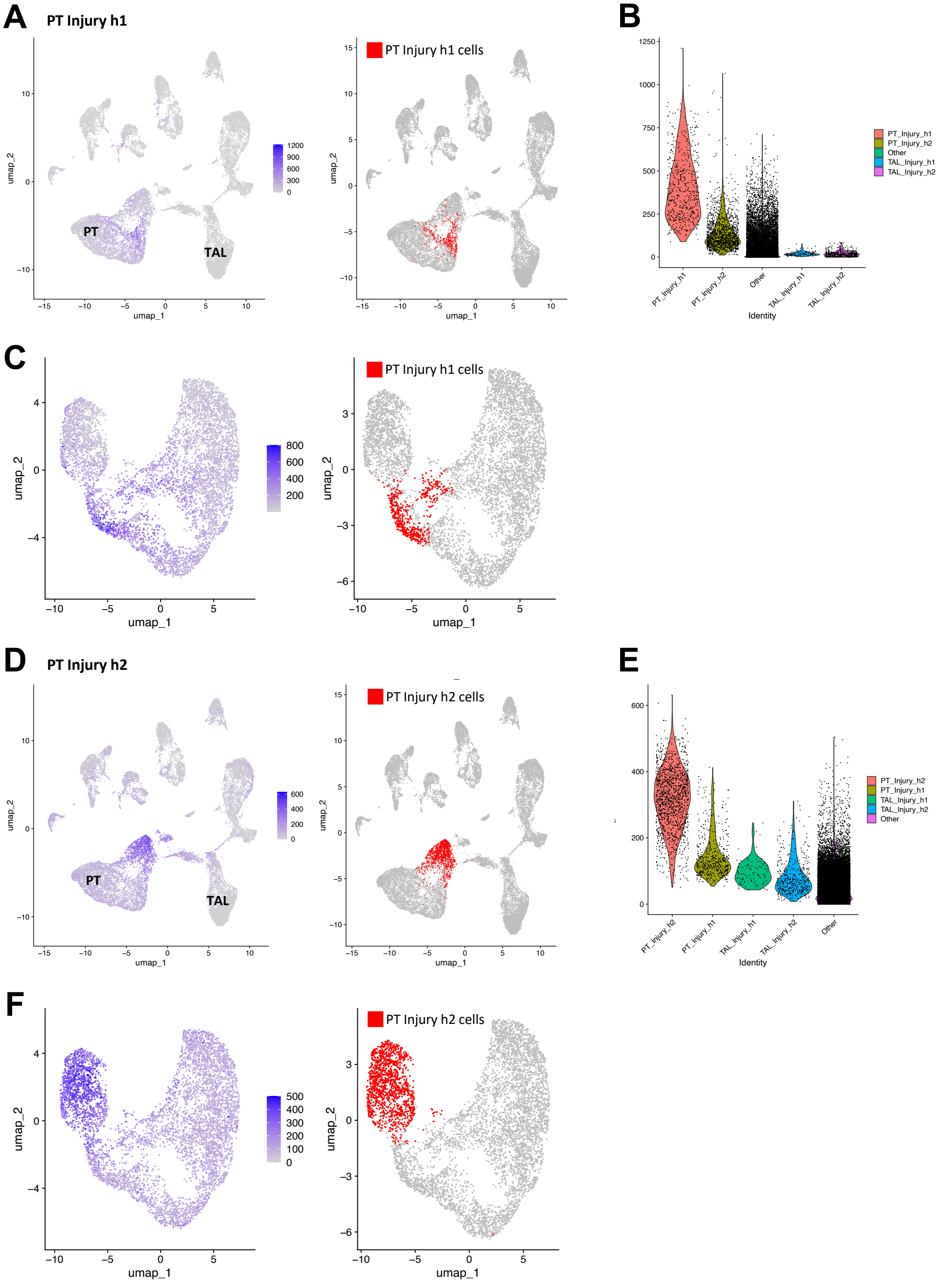

Supplemental Figure S24: Expression patterns for PT gene set scores on human snRNA-seq data. A. *Feature plot for gene set score for PT Injury h1 on all human cells (average counts per million) (left plot), UMAP highlighting PT Injury h1 cells (right plot) and* ***B.*** *violin plot showing average counts per million per cell in all PT and TAL injury clusters and all other cells ("Other").*

C. *Feature plot for gene set score for PT Injury h1 on all human PT cells (left plot) and UMAP highlighting PT Injury h1 cells (right plot).* ***D.-F.*** *Analogous plots for PT Injury h2.*

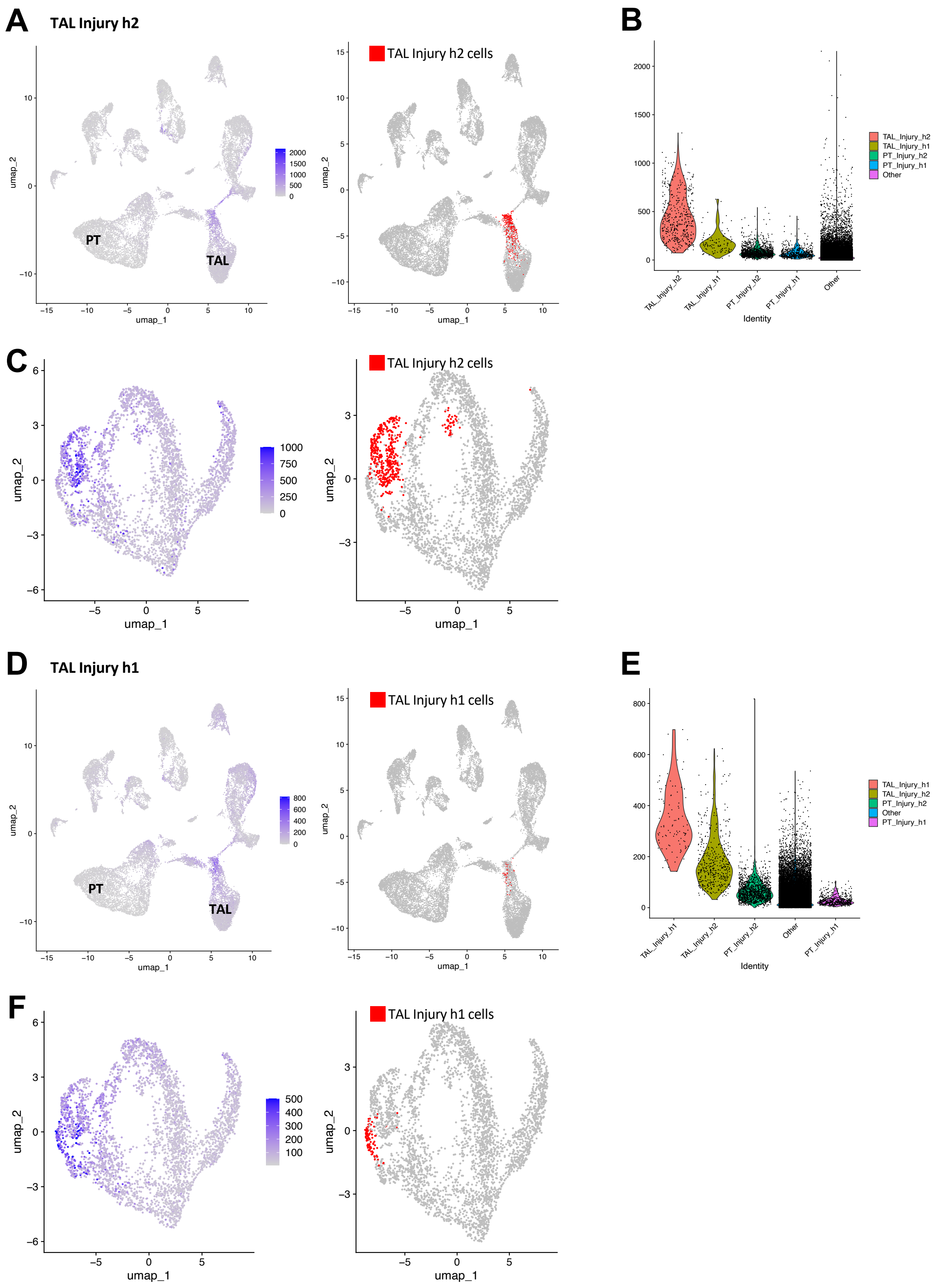

Supplemental Figure S25: Expression patterns for TAL gene set scores on human snRNA-seq data. *Plots are analogous to Fig. S24 for TAL Injury h1 and h2 cells.*

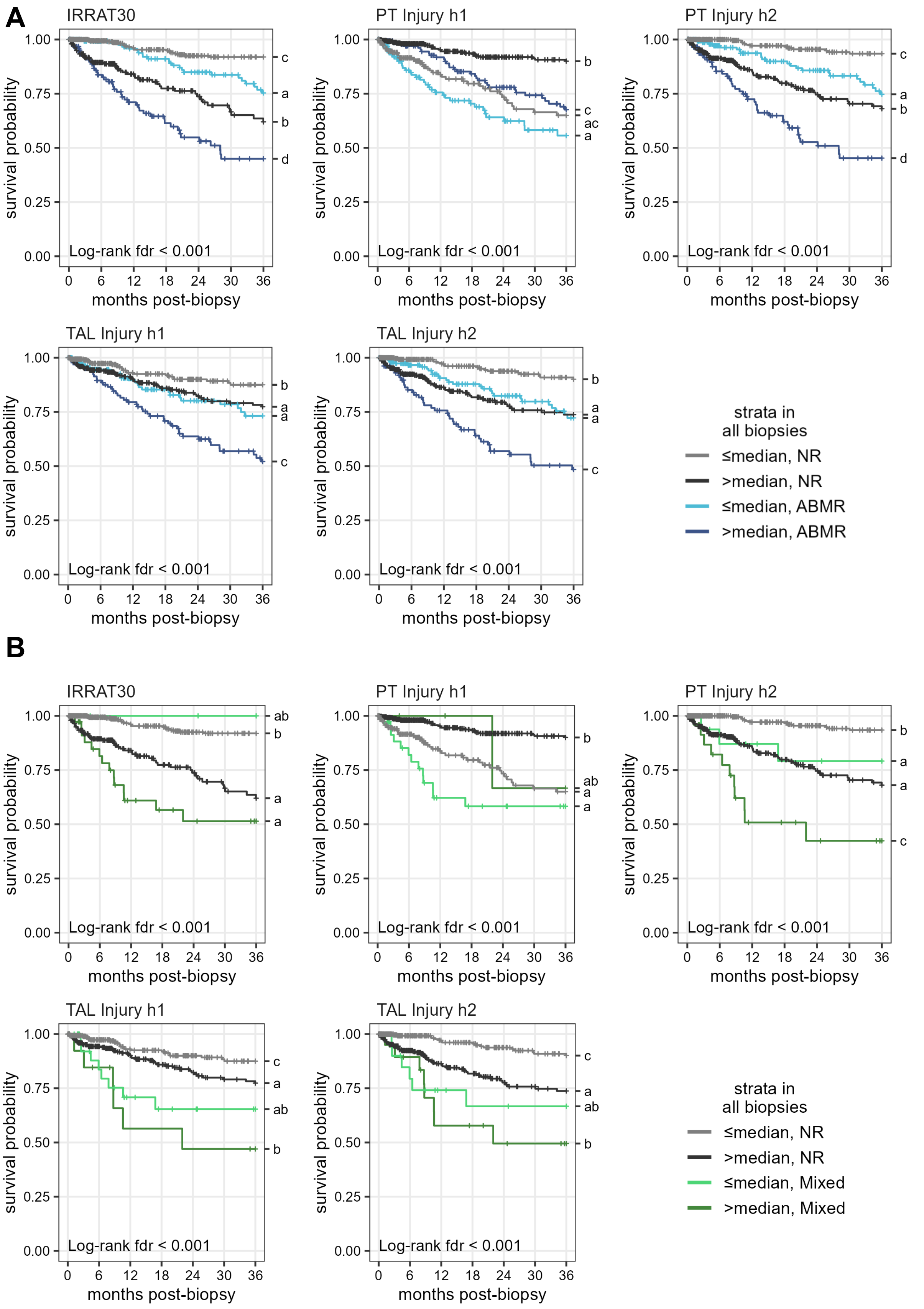
A
IRRAT30
PT Injury h1
PT Injury h2
TAL Injury h1
TAL Injury h2
survival probability
months post-biopsy
Log-rank fdr < 0.001
strata in
all biopsies
≤median, NR
>median, NR
≤median, ABMR
>median, ABMR
B
IRRAT30
PT Injury h1
PT Injury h2
TAL Injury h1
TAL Injury h2
survival probability
months post-biopsy
Log-rank fdr < 0.001
strata in
all biopsies
≤median, NR
>median, NR
≤median, Mixed
>median, Mixed

***Supplemental Figure S26: Impact of gene set score on patients with ABMR and mixed rejection. A**. and **B.** Kaplan Meier curves for allograft survival in cohort of 1061 kidney transplant biopsies (analogous to Fig. 8 for TCMR). Survival is plotted for ABMR (panels under A.), mixed (panels under B.) (TCMR and ABMR) and NR patients below and above the median score of the respective gene set in the full cohort. False discovery rates (fdr) were derived from log-rank test. Identical letters on the right-hand side of the plot indicate no statistical difference. Source data are provided as a Source Data file.*

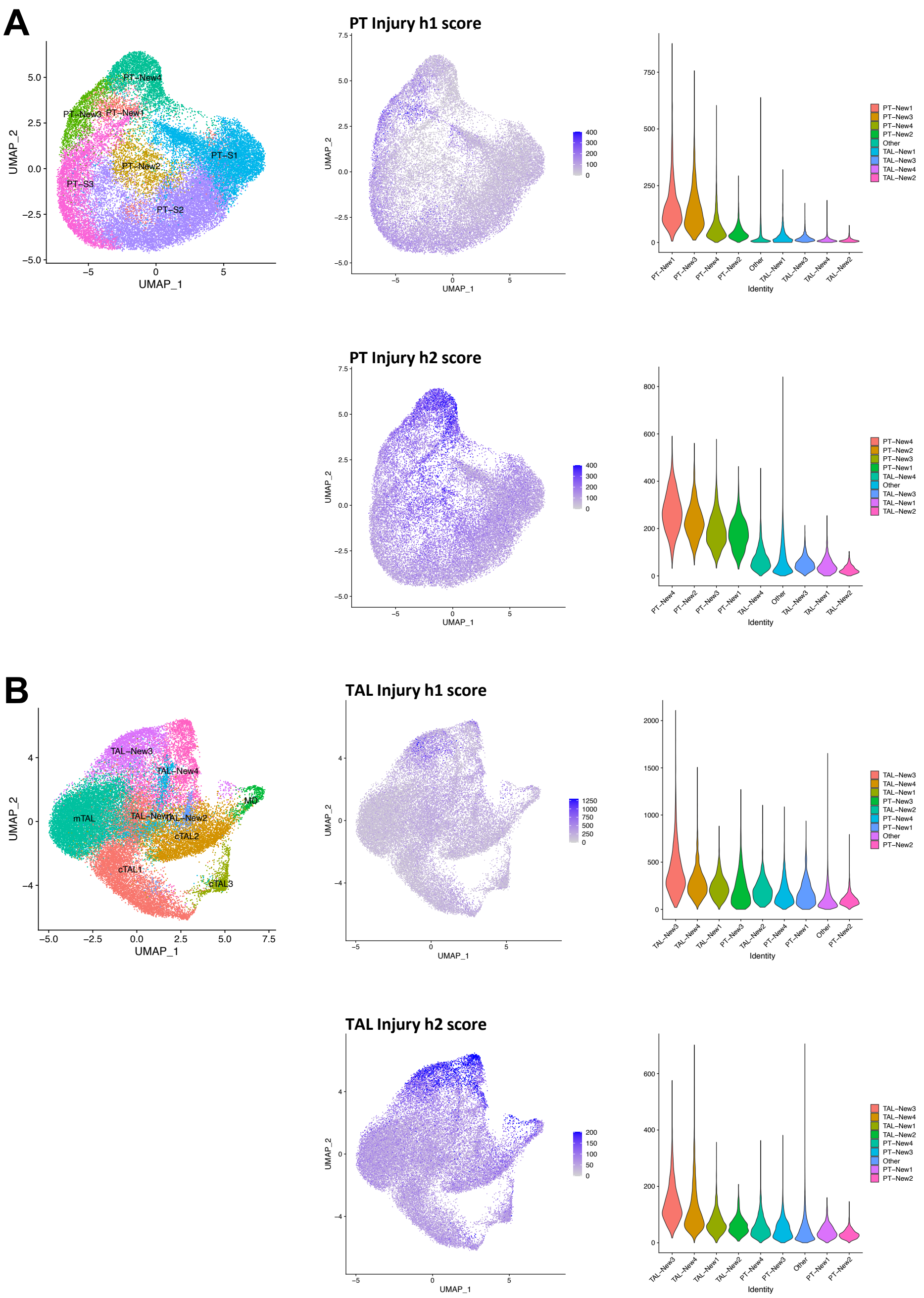

Supplemental Figure S27: Expression patterns for TCMR-induced PT and TAL injury gene set scores on human AKI data. A. *UMAP of PT subclustering as presented in Hinze et al. 2022. This subclustering includes AKI-induced injured PT cell states PT-New 1-4. Feature plots and violin plots are showing the average counts per million score per gene set on the indicated cell populations.* ***B.*** *Analogous plots for AKI-induced TAL cell states TAL-New 1-4.*
