# Supplementary material for: Injured epithelial cell states impact kidney allograft survival after T-cell-mediated rejection
Source: Nat Commun. 2026 Jan 28;17:1060. doi: 10.1038/s41467-026-68397-1 (PMC12852170; doi:10.1038/s41467-026-68397-1)
Supplement: Supplementary file 2 — Description of additional supplementary files [file 41467_2026_68397_MOESM2_ESM.pdf]

Description of Additional Supplementary Files

Supplemental Data 1: Additional custom genes for Xenium spatial transcriptomics.
Supplemental Data 2: Results from differential gene expression TCMR vs. Control.
Supplemental Data 3: Marker genes for PT and TAL Injury cell states used in bulk transcriptomics analyses.
