# Supplementary material for: Injured epithelial cell states impact kidney allograft survival after T-cell-mediated rejection
Source: Nat Commun. 2026 Jan 28;17:1060. doi: 10.1038/s41467-026-68397-1 (PMC12852170; doi:10.1038/s41467-026-68397-1)
Supplement: Supplementary file 6 — Reporting Summary [file 41467_2026_68397_MOESM6_ESM.pdf]

nature portfolio

Corresponding author(s): Philip Halloran, Muhammad Imtiaz Ashraf, Christian Hinze

Last updated by author(s): Nov 21, 2025

Reporting Summary

Nature Portfolio wishes to improve the reproducibility of the work that we publish. This form provides structure for consistency and transparency in reporting. For further information on Nature Portfolio policies, see our Editorial Policies and the Editorial Policy Checklist.

Statistics

For all statistical analyses, confirm that the following items are present in the figure legend, table legend, main text, or Methods section.

n/a	Confirmed	
☐	☒	The exact sample size (*n*) for each experimental group/condition, given as a discrete number and unit of measurement
☐	☒	A statement on whether measurements were taken from distinct samples or whether the same sample was measured repeatedly
☐	☒	The statistical test(s) used AND whether they are one- or two-sided<br>*Only common tests should be described solely by name; describe more complex techniques in the Methods section.*
☐	☒	A description of all covariates tested
☐	☒	A description of any assumptions or corrections, such as tests of normality and adjustment for multiple comparisons
☐	☒	A full description of the statistical parameters including central tendency (e.g. means) or other basic estimates (e.g. regression coefficient) AND variation (e.g. standard deviation) or associated estimates of uncertainty (e.g. confidence intervals)
☐	☒	For null hypothesis testing, the test statistic (e.g. *F*, *t*, *r*) with confidence intervals, effect sizes, degrees of freedom and *P* value noted<br>*Give P values as exact values whenever suitable.*
☐	☒	For Bayesian analysis, information on the choice of priors and Markov chain Monte Carlo settings
☐	☒	For hierarchical and complex designs, identification of the appropriate level for tests and full reporting of outcomes
☐	☒	Estimates of effect sizes (e.g. Cohen's *d*, Pearson's *r*), indicating how they were calculated

Our web collection on statistics for biologists contains articles on many of the points above.

Software and code

Policy information about availability of computer code

Data collection	For snRNA-seq: 10xGenomics cellranger v3.0.2, for Xenium: xeniumranger v2.0, cellbender
Data analysis	custom code is provided on Github: https://github.com/LorenzJahnMHH/TCMR-induced-epithelial-injury-patterns-in-kidney-transplants-main packages used for analysis were: Seurat v5.1.0, SingleR v2.0.0, Scanpy v1.10.1 and v1.11.14, CIBERSORTx platform, squidpy v1.4.1, python v3.10.14, SpatialLeiden v0.2.0

For manuscripts utilizing custom algorithms or software that are central to the research but not yet described in published literature, software must be made available to editors and reviewers. We strongly encourage code deposition in a community repository (e.g. GitHub). See the Nature Portfolio guidelines for submitting code & software for further information.

Data

Policy information about availability of data

All manuscripts must include a data availability statement. This statement should provide the following information, where applicable:

- Accession codes, unique identifiers, or web links for publicly available datasets
- A description of any restrictions on data availability
- For clinical datasets or third party data, please ensure that the statement adheres to our policy

The snRNA-seq and ST data generated in this study have been deposited in the Gene Expression Omnibus database under accession code GSE284742 [https://www.ncbi.nlm.nih.gov/geo/query/acc.cgi?acc=GSE284742]. Source data are provided with this paper. Note that for some graphs derived from snRNA-seq and

spatial transcriptomics data, the underlying source data are not included in the Source Data files due to their volume. These data are available through the referenced GEO dataset. This has been discussed with the editor prior to resubmission.

Research involving human participants, their data, or biological material

Policy information about studies with human participants or human data. See also policy information about sex, gender (identity/presentation), and sexual orientation and race, ethnicity and racism.

Reporting on sex and gender	Sex data is included in Suppl. Table S1, gender data was not collected. For bulk transcriptomics, individual sex data, whenever available, is provided in the source data files.
Reporting on race, ethnicity, or other socially relevant groupings	Other population characteristics apart from what is shown in Suppl. Table S1 or in the source data are not presented.
Population characteristics	For snRNA-seq: A total of 6 patient samples were included coming from 4 male and 2 female participants (age range 49-77, see Suppl. Table S1). Sex assigned at birth was extracted from clinical records. No gender identity data were collected. Sex-based analyses were not carried out because sample size in each subgroup was insufficient for meaningful stratification. For bulk transcriptomics: 1. For gene set scores and allograft survival: 1061 patients from 605 male, 353 female and 103 unreported sex participants (age range 2-92 years). Sex assigned at birth was voluntarily provided by each patient's clinician. No gender identity data were collected. For single cell deconvolution: 3858 biopsies from 3210 patient samples were included coming from 1019 male, 600 female and 1591 unreported sex participants (age range 2-92 years). Sex assigned at birth was voluntarily provided by each patient's clinician. No gender identity data were collected.
Recruitment	snRNA-seq: Samples were chosen from the Hannover Medical School protocol biopsy program for the presence of TCMR or no pathologies (stable allograft) and availability of kidney biopsy core in RNAlater. Final 6 patient samples were randomly selected. Bulk transcriptomics: We included a total of 5086 kidney transplant biopsies. 3570 biopsies from 3995 patients were collected through International Collaborative Microarray Extension Study (INTERCOMEX; NCT01299168) and Trifecta-Kidney (NCT04239703) clinical trials and MMDx-Kidney sub-studies. An additional 1516 biopsies, included from MMDx service laboratory in Portland, OR (Kashi Laboratories, submitted as anonymized files for this study), were processed for MMDx.
Ethics oversight	snRNA-seq: Ethics committee of Hannover Medical School, bulk transcriptomics: informed consent per institutional review board review at each local center and approved in Edmonton by the University of Alberta

Note that full information on the approval of the study protocol must also be provided in the manuscript.

Field-specific reporting

Please select the one below that is the best fit for your research. If you are not sure, read the appropriate sections before making your selection.

☒ Life sciences ☐ Behavioural & social sciences ☐ Ecological, evolutionary & environmental sciences

For a reference copy of the document with all sections, see nature.com/documents/nr-reporting-summary-flat.pdf

Life sciences study design

All studies must disclose on these points even when the disclosure is negative.

Sample size	The nature of our study is purely exploratory. For snRNA-seq, from our experience, 3 samples per group for single cell sequencing data is usually sufficient. No separate power analysis was performed. For bulk transcriptomics, we included all available biopsies.
Data exclusions	No data were excluded from the study.
Replication	We provide biological replicates for all experiments which confirmed all results.
Randomization	No randomization.
Blinding	Investigators were not blinded during acquisition and analysis.

Reporting for specific materials, systems and methods

We require information from authors about some types of materials, experimental systems and methods used in many studies. Here, indicate whether each material, system or method listed is relevant to your study. If you are not sure if a list item applies to your research, read the appropriate section before selecting a response.

Materials & experimental systems

n/a	Involved in the study	
☐	☒	Antibodies
☒	☐	Eukaryotic cell lines
☒	☐	Palaeontology and archaeology
☐	☒	Animals and other organisms
☐	☒	Clinical data
☒	☐	Dual use research of concern
☒	☐	Plants

Methods

n/a	Involved in the study	
☒	☐	ChIP-seq
☒	☐	Flow cytometry
☒	☐	MRI-based neuroimaging

Antibodies

Antibodies used	All antibodies are listed in detail in the methods section (Tables 1 and 2)
Validation	All antibodies used in this study were validated for the respective species and applications as indicated by the manufacturer's datasheets, confirmed in published literature, and further supported by experimental data provided in the Methods section and Supplementary Figures. Moreover, most of the antibodies are widely used in our lab and have been validated in several independent studies.

Animals and other research organisms

Policy information about studies involving animals; ARRIVE guidelines recommended for reporting animal research, and Sex and Gender in Research

Laboratory animals	C57BL/6 and BALB/c mice.
Wild animals	The study did not involve wild animals.
Reporting on sex	We only used male mice.
Field-collected samples	The study did not involve field-collected samples.
Ethics oversight	Landesamt für Gesundheit und Soziales, Berlin, Germany.

Note that full information on the approval of the study protocol must also be provided in the manuscript.

Clinical data

Policy information about clinical studies
All manuscripts should comply with the ICMJE guidelines for publication of clinical research and a completed CONSORT checklist must be included with all submissions.

Clinical trial registration	not applicable
Study protocol	not applicable
Data collection	not applicable
Outcomes	not applicable

Plants

Seed stocks	not applicable
Novel plant genotypes	not applicable
Authentication	not applicable
